# Unveiling plant protein astringency perception through neural and cellular responses

**DOI:** 10.1038/s41598-025-23836-9

**Published:** 2025-12-04

**Authors:** Ben Kew, Melanie Rose Burke, Markus Stieger, Yunqing Wang, Christine Boesch, Melvin Holmes, Anwesha Sarkar

**Affiliations:** 1 Food Colloids and Processing Group, School of Food Science and Nutrition, University of Leeds, Leeds, LS2 9JT UK; 2 National Alternative Protein Innovation Centre (NAPIC), Leeds, UK; 3 School of Psychology, University of Leeds, Leeds, LS2 9JT UK; 4 Division of Human Nutrition and Health, Wageningen University, PO Box 17, Wageningen, 6700 AA The Netherlands; 5 Nutritional Sciences and Epidemiology Group, School of Food Science and Nutrition, University of Leeds, Leeds, LS2 9JT UK

**Keywords:** Psychology, Cell biology

## Abstract

While consumption of plant proteins providing nutritional, social and climate solutions to feed a growing population in a healthy and sustainable manner is unquestionable, astringency remains a persistent yet underestimated organoleptic challenge. This longstanding issue, contributing to a sensation of dryness, serves as a major bottleneck in the development of plant-based foods with broad consumer acceptance. Here, we investigate experimentally the origin of astringency in plant proteins probing sensory, neural, and cellular responses. Strikingly, by combining sensory profiling with functional near-infrared spectroscopy (fNIRS) and saliva and mucin-coated oral epithelium-mimicking cell lines, we identify that plant protein astringency demonstrates a distinct prefrontal cortex neural response and is associated with salivary mucin-binding, drawing parallels to tannin-like responses. Our mechanistic dissection fuelled by in vitro, in vivo, and ex vivo experimental studies pinpoints direct quantitative evidence uncovering previously uncharacterised astringency of plant proteins, paving the way for solving the sensorial challenges in the era of transitioning towards eco-friendly plant protein-based foods.

**Supplementary Information:**

The online version contains supplementary material available at 10.1038/s41598-025-23836-9.

## Introduction

The concurrent prevalence of chronic disease, partly driven by excessive meat consumption, alongside the critical challenge of substantial greenhouse gas (GHG) emissions from animal-based food production, is exerting escalating pressures on global food systems. Recently it was found that over one-sixth of the total anthropogenic GHG emissions occurs solely from animal protein industry^1^. Additionally, excessive animal meat consumption, particularly processed and red meat significantly contributes to increased mortality and disease risk^2,3^. These findings underscore the urgent need to safeguard planetary health, which requires a transition away from the more environmentally detrimental animal protein sources to alternative plant protein-rich foods. Nevertheless, plant proteins face significant sensory hurdles; including off-flavours, dry mouthfeel, and astringency^4–8^, which bottlenecks effective incorporation into food products and repeated consumption by consumers. In particular, the astringent perception remains to be a major barrier and a distinct longstanding challenge towards adoption of plant proteins^9^. This sensation, described as the most enigmatic tactile percept, in other words, mechanosensation^10^ results in oral dryness, puckering and powdery or gritty feeling on the tongue and throughout the oral cavity.

Astringency is defined by the American Society for Testing Materials as “the complex of sensations due to shrinking, drawing or puckering of the epithelium as a result of exposure to substances such as alums or tannins”^11^. The phenomenon caused by phenolic compounds such as tannins and catechins present in tea, coffee, wines and, certain unripe fruits is relatively well studied^12^. In addition to phenolic compounds, proteins^13–15^, polymers^16^, and multivalent cations^17^ have also been reported to evoke an astringent mouthfeel. Astringency is often hypothesised to be associated with failure of salivary lubrication where astringent compounds bind and precipitate certain salivary proteins such as proline-rich proteins (PRPs)^18,19^ and/or disrupt the mucosal pellicle coating the oral epithelium^20–22^, although not all astringent compounds aggregate salivary proteins. An alternative conjecture^23^ is aggregation and possible dissociation of tethered transmembrane mucin (MUC1) from the mucosal pellicle, and consequent increase of the friction forces at the surface of the oral mucosa which causes astringency in phenolic compounds. The pioneering work by Schöbel et al. (2014)^24^ involving rodent and human models demonstrated that the astringency perception by phenolic molecules requires intact trigeminal neural circuits^10^. Despite multiple possible mechanisms having been suggested in phenolics, the complex molecular, cellular and neural mechanisms underpinning astringency caused by plant proteins, which are inherently aggregated^25^, remains largely unknown. What is key is to first understand the fundamental causes of astringency in plant proteins before implementing processing strategies to develop plant-based sustainable foods that are non-astringent and acceptable to consumers.

Whilst astringency is frequently being reported in plant protein food products^6,26–29^, nothing is yet known about whether astringency is indeed a percept in isolated plant proteins. Expectedly, the current paradigm in astringency perception in plant foods is largely restricted to human-derived sensory ratings in real foods. This investigation utilises model systems enabling the isolated investigation of protein-related tactile responses^30–32^ where recent study^33^ demonstrate a dominant role of plant proteins in Additionally model WPI protein solutions at neutral pH has been assessed previously providing valuable insights into astringency across pH with trained panellists finding little and no perception of astringency recorded at neutral pH providing an interesting comparison in our study^34^. Owing to the complexity and diversity of biological mechanisms involved, it is essential to adopt a comprehensive, multiscale approach to understand the underlying mechanism of astringency of plant proteins which we have tested by combining sensorial perception with neural and cellular study maintaining a model samples throughout. Previous neural studies have typically utilised functional magnetic resonance imaging (fMRI) for both texture and taste research including viscosity^35,36^, umami^37^, oral fat^35^, sweetness and pungency^38,39^ that have been explored. Although fMRI provides spatial resolution in the brain, it lacks temporal resolution^40^ which impedes its reliable use in astringency perception during oral processing that ranges from few milliseconds to sub-minute ranges. Furthermore, fMRI is sensitive to head motion, requiring that the human subject is immobile, laying on their back which limits the neural assessment during natural eating behaviour. Recent advances in functional near-infrared spectroscopy (fNIRS) serve as a more suitable approach that measures haemoglobin changes via absorbed near infrared light where both oxyhaemoglobin (HbO) and deoxyhaemoglobin (HbR) changes are determined. fNIRS proves a higher temporal resolution whilst offering flexibility, full movement and mobility by simply requiring the user to wear a cap. Previous studies have investigated taste perception using fNIRS and found significant dorsolateral prefrontal cortex activity being involved in secondary gustatory responses^41,42^ when assessing preference and taste between two plant based milks^43^. However, no previous study has used fNIRS to identify whether the astringency of plant proteins has a similar neural basis to those of phenolic compounds, which is a focus of the current study. Finally, little is known about how plant proteins interact with human saliva tethered to mucinous oral cells, and we hypothesize a mechanism similar to those in tannins on salivary precipitation that can explain astringency mechanism^44,45^ at a cellular scale.

**Fig. 1:**
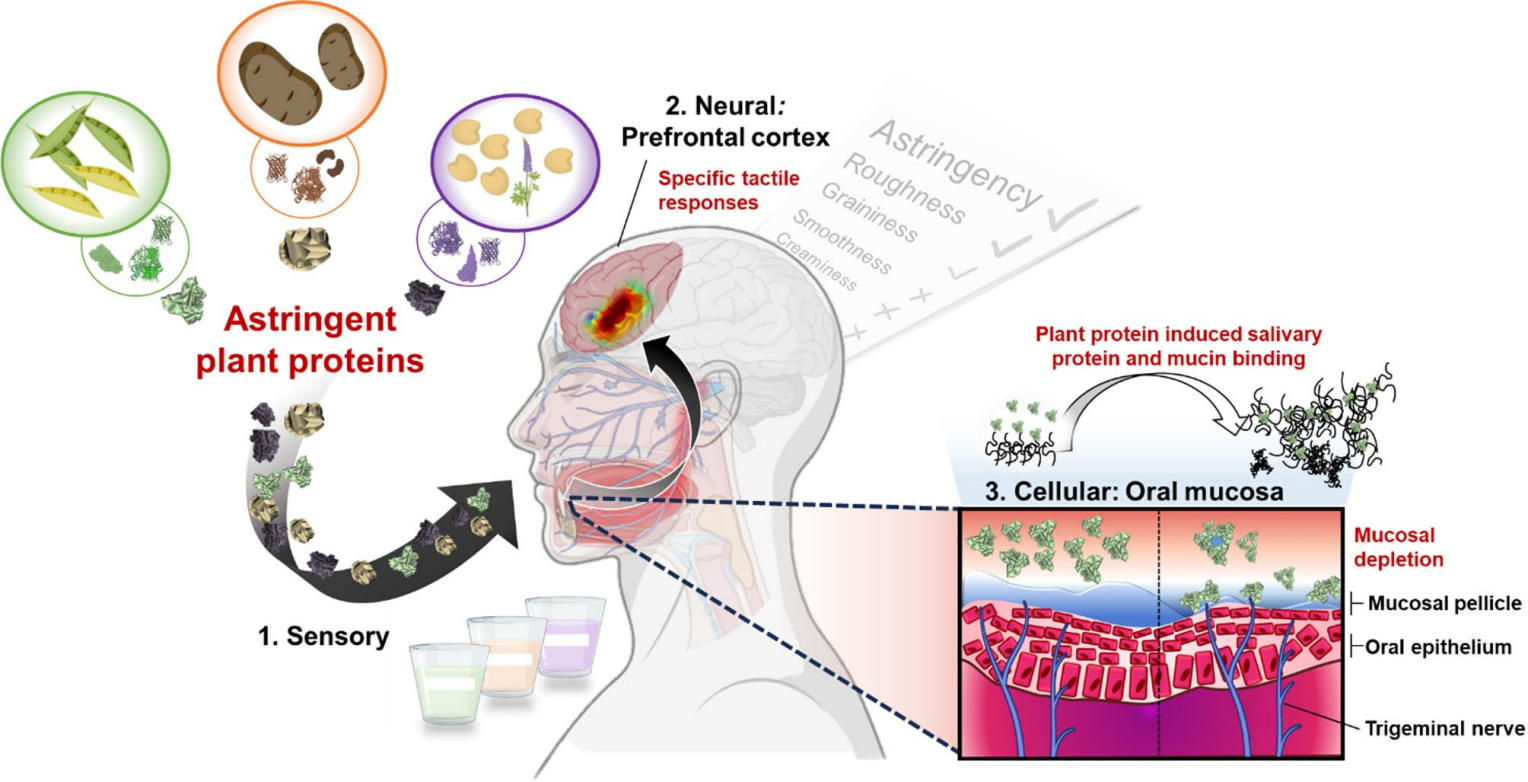
Illustrative overview of methodologies used to unveil the sensory, neural and cellular response to plant proteins. A combination of sensory trial with untrained human participants (*n* = 100) using Rate-All-That-Apply, (RATA), a study of neural response in prefrontal cortex of human participants (*n* = 34) measured using Functional Near-Infrared Spectroscopy (fNIRS) and cellular response measured using saliva-coated oral epithelium based TR146-MUC1 cell lines was used to unveil plant protein astringency mechanisms. The astringency is uniquely detected in the prefrontal cortex of the brain leading to negative tactile responses which resulted from physical binding of plant proteins with saliva bound to oral epithelium, inducing astringency perception. Ethical approval was obtained from the University of Leeds (MEEC 16–046 and PSYC-475) by the Faculty Ethics Committee, University of Leeds, UK.

Herein, we have designed a unique combinatorial approach of sensory, neural and cellular methods to elucidate the astringency mechanism of isolated plant proteins (Fig. 1) Combining astringency ratings from participants (*n* = 134 total participants) with fNIRS and saliva-coated mucinous cell lines, we specifically find that isolated plant proteins are astringent and such astringency has a cellular and neural basis resembling that of tannin-mediated mucin interaction. In summary, these results provide a comprehensive in vitro, in vivo and ex vivo characterisation decoding the underlying mechanism and impacts of astringency and set the stage for design principles in the next generation of plant protein-rich foods, thus accelerating the much-needed transition towards a plant-based sustainable food system.

## Results

Two legume proteins, pea protein concentrate (PPC), lupin protein isolate (LPI), along with a tuber protein i.e. potato protein isolate (PoPI) were assessed, reflecting their interest by both scientific and industrial communities due to enhanced sustainability features and functional properties^46–49^. The extent of astringency was determined comparing plant protein type and at low to high protein concentrations (5 wt% − 15 wt% total protein). Scaled quantitative ratings on the aforementioned plant protein were collected using Rate-All-That-Apply (RATA) sensory analysis^50^. Given that astringency originates from tactile sensation, we also examined its association with other textural attributes including mouthcoating, roughness, and graininess while considering its temporal progression over time by assessing after-feel (AF) (see Supplementary Table 1 for definitions of sensory terms) to capture lasting or cumulative textural responses^51,52^.

### Tactile response in model plant protein formulations

**Fig. 2:**
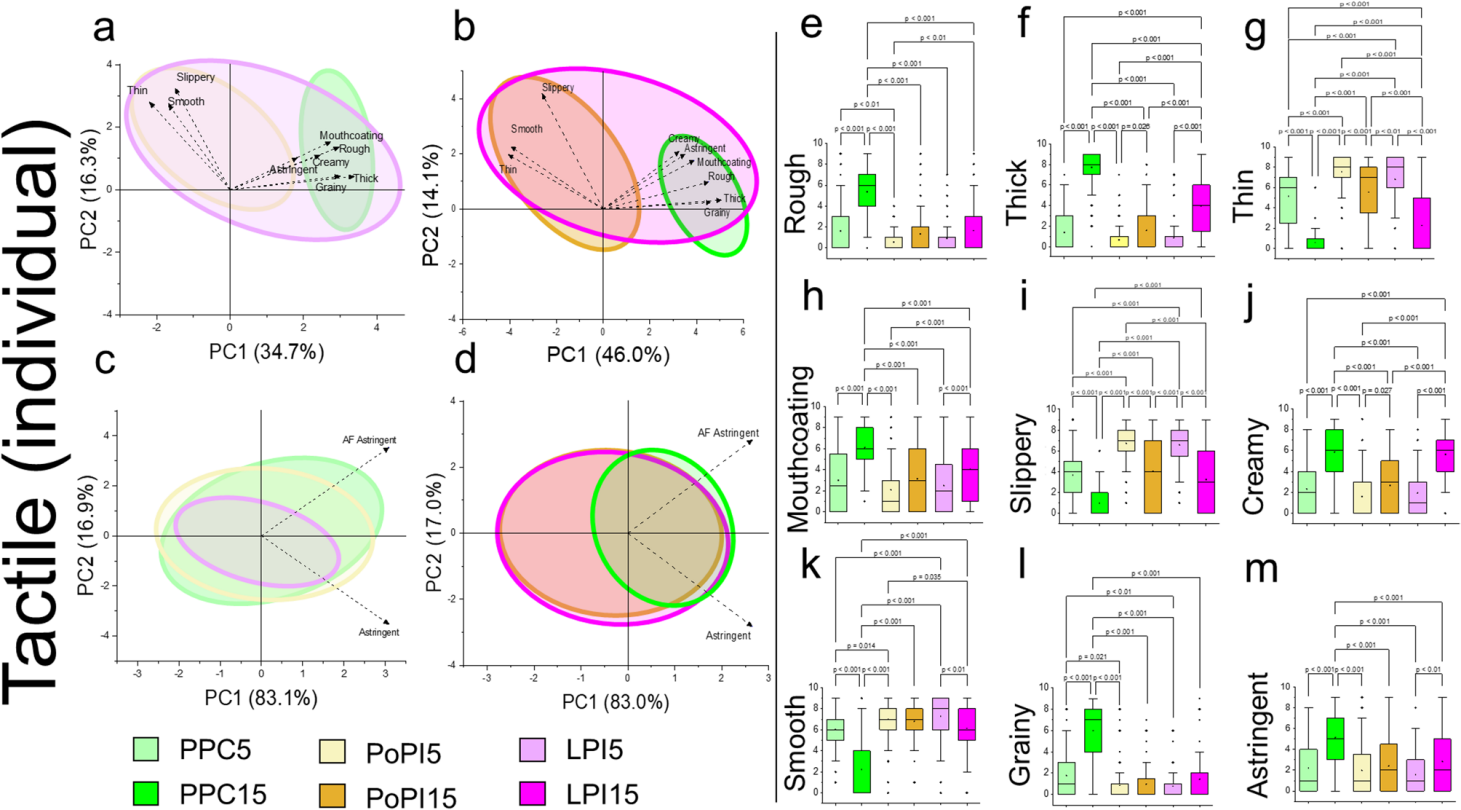
Mouthfeel characteristics of model plant protein formulations individually at low and high concentrations. Principle component analysis (PCA) of the mouthfeel attributes of model plant protein formulations containing plant proteins individually at (**a**) 5 wt% total protein (**b**) 15 wt% total protein and corresponding astringency attributes at (**c**) 5 wt% total protein (**d**) 15 wt% total protein, respectively. Tactile ratings from Rate-All-That-Apply (RATA) are shown at 5 wt% and 15 wt% total protein for pea protein concentrate (PPC5, PPC15), potato protein isolate (PoPI5, PoPI15), lupin protein isolate (LPI5, LPI15) in (**e-m**) for nine different tactile attributes. Results are presented as bar and whisker plots with interquartile range, minimum and maximum plotted (*n* = 100 participants). Pairwise comparisons were made between type (PPC, PoPI, LPI) and protein concentration (5–15 wt% total protein) for each of the attributes applying Bonferroni correction. Ethical approval was obtained from the University of Leeds (MEEC 16–046 and PSYC-475) by the Faculty Ethics Committee, University of Leeds, UK.

Principal component analysis (PCA) revealed distinct textural differences between plant proteins at 5 wt% (Fig. 2a) and 15 wt% total protein (Fig. 2b). Noteworthy, PCA is separated based on potato protein (PoPI5 or PoPI15) that is thin, slippery and smooth compared to pea protein (PPC5 or PPC15) being thick, grainy, rough, mouthcoating and astringent whilst lupin protein (LPI5 or LPI15) reflects a mixture of tactile attributes (Fig. 2a-b). This demonstrates that the protein type did influence the tactile attributes in model protein formulations whilst concentration made the distinction between the protein types more obvious, which is also apparent for all attributes (Supplementary Fig. 1a-b). To rule out the effects of taste and flavour, sensory perceptions of the samples were matched at 5 wt% and 15 wt% to which PCA remains indistinct between the type of protein at 5 and 15 wt% protein (Supplementary Fig. 2). Isolating just astringency and astringency after feel (AF) values in PCA (Fig. 2c-d), we reveal remarkably little PCA separation between protein concentrations, with the exception of pea protein (Fig. 2d). This data revealed that astringency was rather an omnipresent attribute in model plant protein formulations.

With tactile response revealing the most significant sensory characteristics for each protein, we then assessed the absolute tactile value in depth. For low protein formulations (5 wt% total protein), PoPI5 and LPI5 were perceived similarly in tactile attributes such as roughness, thickness, thinness, mouthcoating, slipperiness, smoothness, graininess and astringency (Fig. 2e-m) (*p < 0.05*).

However, these proteins were distinct to PPC5, with pea protein coding significantly lower in thin perception (Fig. 2g, p *< 0.001*), slipperiness (Fig. 2e, p *< 0.001*), smoothness (Fig. 2g, *p* < 0.014), and consequently coding significantly higher in roughness (Fig. 2e, p *< 0.01*) and graininess (Fig. 2l, p *< 0.022*). As one might expect, for higher protein concentrations of 15 wt% protein, all formulations had increased viscosity leading to greater thickness (Fig. 2f) and less thinness (Fig. 2g) perception reflected in all proteins (Fig. 2f-g, p *< 0.027*). Of more importance, increasing protein concentration resulted in significant reductions in slipperiness (Fig. 2i, p *< 0.001*) but increased creaminess (Fig. 2j, p *<  0.028*) for all protein types. Strikingly, the influence of type of protein becomes more apparent at 15 wt% usage levels. For instance, PoPI15 was rated the least thick (Fig. 2f, p *< 0.001*) and also the least creamy (Fig. 2j, p *< 0.001*) of the proteins sampled, changing little compared to lower protein concentration (PoPI5). In other words, potato protein showed limited concentration dependence in tactile responses. Comparing with other 15 wt% proteins, PoPI15 shared sensory similarities with LPI15 ratings in most tactile ratings except for mouthcoating (Fig. 2h), slipperiness (Fig. 2i), astringency (Fig. 2m), whilst coding lower in roughness (Fig. 2a) and graininess (Fig. 2l) (*p > 0.05*) and with LPI15 rated thicker (Fig. 2f, *p* < 0.001) than PoPI15.

Remarkably, PPC15 stood out amongst the proteins where it was rated the most rough (Fig. 2e, p *< 0.001*), highest mouth coating (Fig. 2h, p *< 0.001*), being grainy (Fig. 2l, p *< 0.001*) and astringent (Fig. 2m, p *< 0.001*) whilst concurrently being lowest in slipperiness (Fig. 2i, p *< 0.001*) and smoothness (Fig. 2k, p *< 0.001*). Nevertheless, to our surprise, PPC15 was interestingly creamy (Fig. 2j), likely as a result of being the most viscous sample (Fig. 2f, p *< 0.001*). Creaminess is a complex textural attribute combining both rheological and surface textural responses that is long known to be a desired attribute (Kokini et al., 1977; Elmore et al., 1999), we speculate in these plant proteins (devoid of any fat) creaminess perception is dominated primarily by thickness. Graininess on the other hand is often an undesired attribute associated with the formation of large particles in the mouth, which might result from an oral interaction with saliva that leads onto roughness and loss of slipperiness and smoothness^53^. PPC15 at higher concentrations significantly contributed to graininess that was four-to-six times higher than that with LPI15 and PoPI15 (Fig. 2l, p *< 0.001*), which is in line with our previous in vitro findings where pea protein has demonstrated particle-particle aggregation and increased particle size in dispersion^54,55^. Such poor performance of PPC15 may be due to its significantly highly rated astringency (Fig. 2m, p *< 0.001*) which doubles in intensity compared to PPC5, highlighting the influence of concentration dependency of astringency, particularly in pea protein. Consistently, astringency was prevalent for all protein types and concentration and were coded synergistically with mouthcoating, roughness, graininess, thick and also non-intuitively creaminess (Fig. 2m), whilst increasing protein concentration increased the tactile issues where pea protein (PPC15) particularly stood out.

**Fig. 3:**
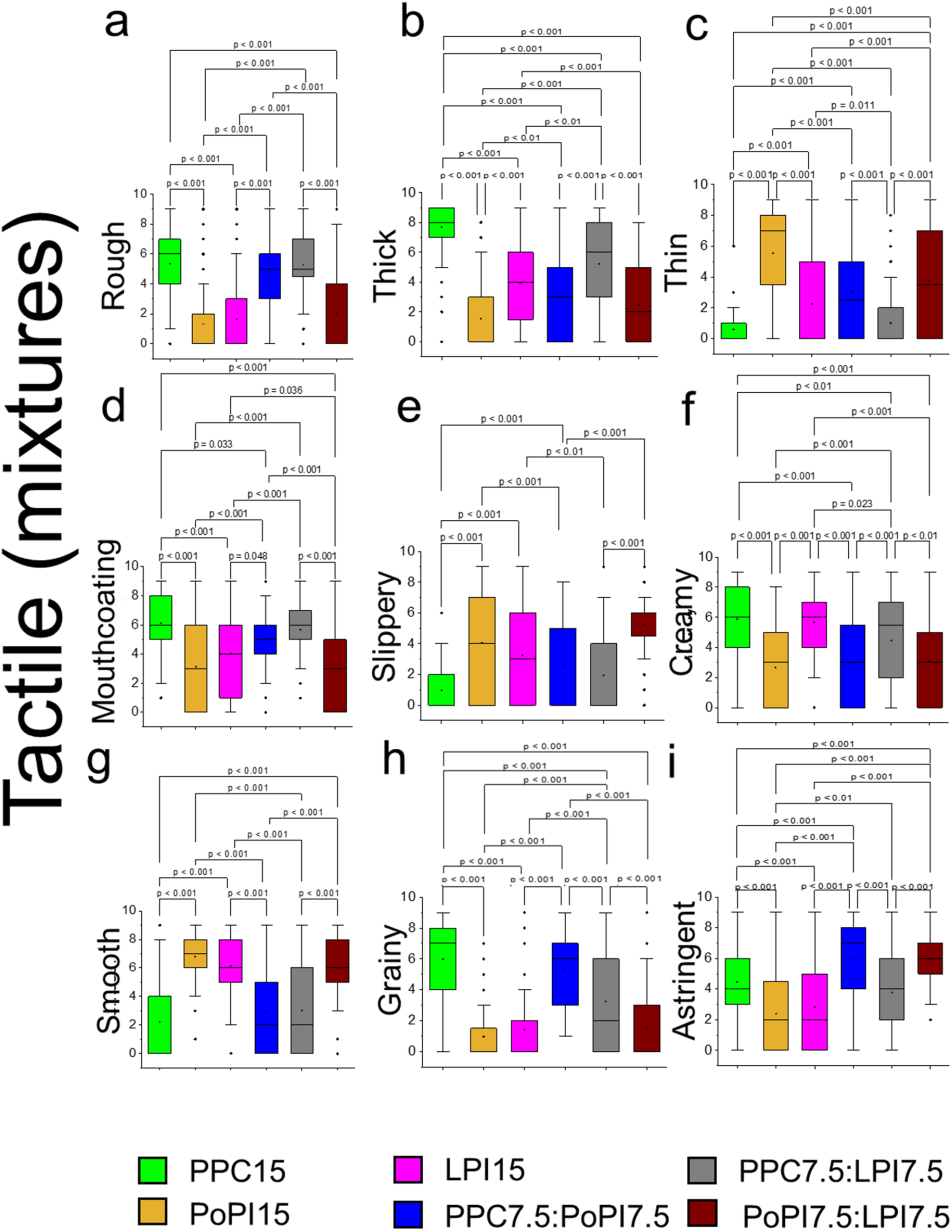
Tactile characteristics of mixed protein formulations compared with individual proteins at higher protein levels. Tactile ratings from Rate-All-That-Apply are shown at 15 wt% total protein for pea protein concentrate (PPC15), potato protein isolate (PoPI15), lupin protein isolate (LPI15) and 1:1 binary protein mixtures (PPC7.5:PoPI7.5, PPC7.5:LPI7.5, PoPI7.5:LPI7.5) (**a-i**) obtained using Rate-All-That-Apply (RATA) in nine different tactile attributes. Results are presented as bar and whisker plots with interquartile range, minimum and maximum plotted (*n* = 100 participants). Pairwise comparisons were made between type (PPC, PoPI, LPI, PPC-LPI, PPC-PoPI, PoPI-LPC) for each of the attributes applying Bonferroni correction. Ethical approval was obtained from the University of Leeds (MEEC 16–046 and PSYC-475) by the Faculty Ethics Committee, University of Leeds, UK.

Having analysed the astringency perceptions of individual proteins, we tested 1:1 binary protein mixtures (15 wt% total protein, PPC7.7:PoPI7.5, PPC7.5:LPI7.5, PoPI7.5:LPI7.5). It is widely recognised individual plant proteins often lack one or more essential amino acids; for example, legume proteins typically have lower levels of methionine, cysteine, and tryptophan, while cereal proteins are deficient in lysine^56–58^. Protein complementation is often employed to correct this imbalance; therefore, we evaluated potential synergistic or adverse effects on astringency and tactile responses in comparison with model formulations containing individual proteins. Except for a synergistic improvement in slipperiness observed in PoPI7.5:LPI7.5 (Fig. 3e, p *< 0.001*), the protein mixtures generally displayed typical behaviour, closely resembling or scoring between the individual proteins across most attributes (Fig. 3a-i). However, we observed a detrimental effect on astringency for all mixtures as they were rated significantly more astringent for instance, PoPI mixtures (PPC7.5:PoPI7.5, PoPI7.5:LPI7.5) compared to their individual counterparts (Fig. 3i, p *< 0.001*).

Lastly from a flavour perspective, (Supplementary Fig. 3a and 3e, *p* *> 0.05*), we could only detect noticeable differences when potato protein was combined with either lupin or pea (PPC7.7:PoPI7.5, PoPI7.5:LPI7.5) where a significant increase in off flavour occurs (Supplementary Fig. 3 d, *p* *> 0.01*), while no significant differences are found in other flavours (Supplementary Fig. 3b-c, *p* *> 0.05*). Taken together, these sensory results strongly suggest that astringency is not specific to a particular plant protein and that a combination of plant proteins cannot prevent astringency perception.

### Temporal tactile response of model plant protein formulations

**Fig. 4:**
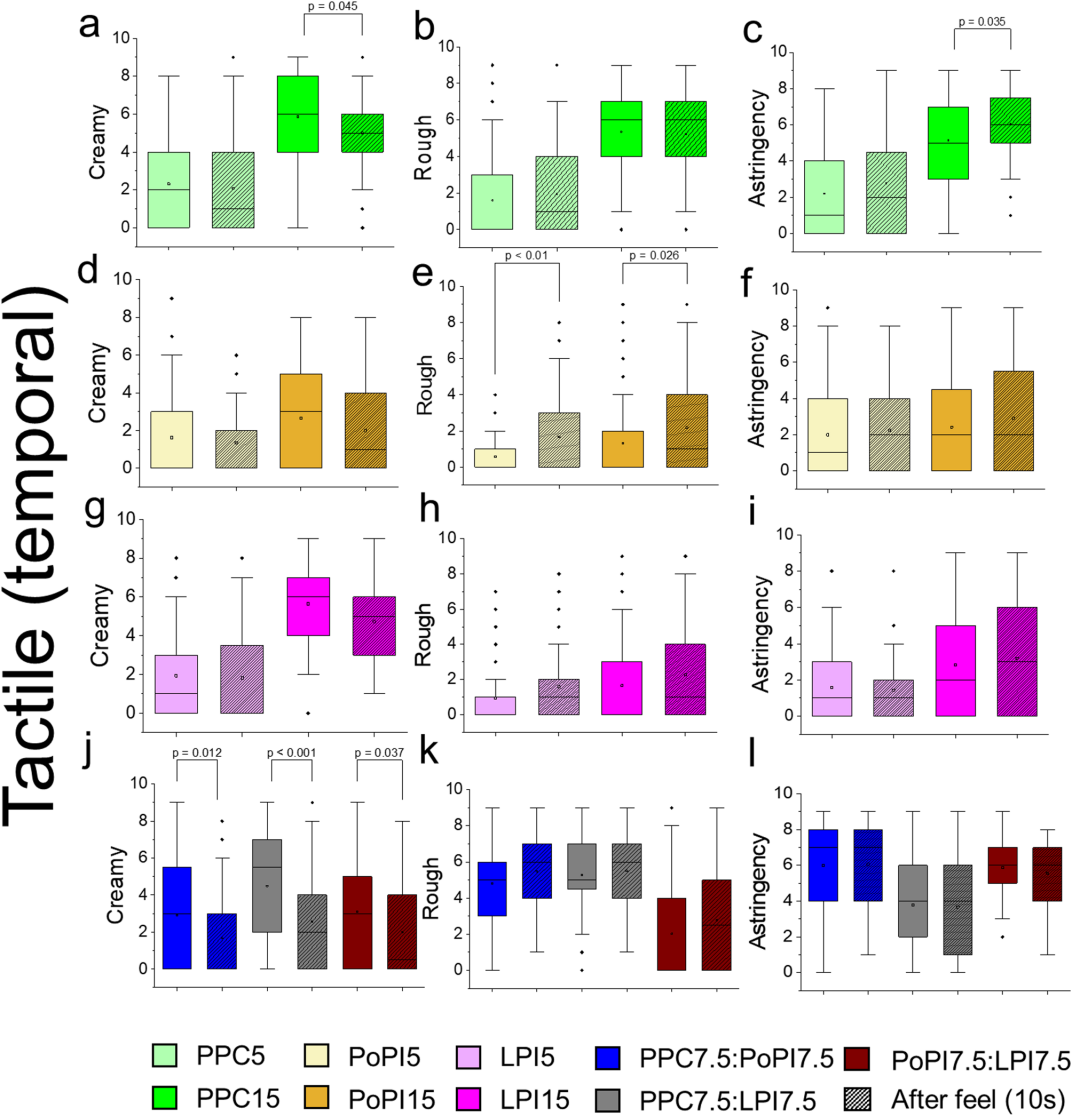
Temporal tactile responses of model plant protein formulations. Tactile ratings during and 10 s post consumption from Rate-All-That-Apply (RATA) are shown for pea protein concentrate (PPC15), potato protein isolate (PoPI15), lupin protein isolate (LPI15) and mixtures at 1:1 weight ratio (PPC7.5:PoPI7.5, PPC7.5:LPI7.5, PoPI7.5:LPI7.5) (**a-i**) at 15 wt% total protein in nine different tactile attributes. Results are presented as bar and whisker plots with interquartile range, minimum and maximum plotted (*n* = 100 participants). Pairwise comparisons were made between same proteins but for each of the attributes applying Bonferroni correction. Ethical approval was obtained from the University of Leeds (MEEC 16–046 and PSYC-475) by the Faculty Ethics Committee, University of Leeds, UK.

With astringency being a prominent feature for all tested plant proteins, we hypothesize a lingering temporal response may exist and lead to subsequent tactile deterioration as a potential cause for commonly observed product rejection^59^. A pairwise comparison was conducted on the initial vs. after 10 s post consumption ratings of creaminess, roughness and astringency for 5 wt%, 15 wt% total protein and mixtures (Fig. 4a-l). For PPC15 post consumption, creaminess significantly decreased (Fig. 4a, p *< 0.05*) and astringency increased (Fig. 4c, p *< 0.001*) which suggests this temporal mouth-interacting effect with pea protein causing an instantaneous creaminess but prolonged undesirable sensorial astringency upon consumption. For potato protein (PoPI5 and PoPI15), we observed a significant increase in roughness post consumption irrespective of concentration (Fig. 4e, p *< 0.027*). This may initially be masked by high levels of slipperiness (Fig. 2i), but then proteins left behind plausibly interact with saliva precipitating lubricating salivary proteins causing roughness causing high astringency ratings (Fig. 4f). Strikingly for mixtures, there is a loss of creaminess for all proteins (Fig. 4j, p *< 0.037*), which is not surprising as creaminess most likely resembled initial thickness (Fig. 2g and f), however for after-feel, their high astringency and roughness (Fig. 4k-l) persisted. We have shown that this effect in plant proteins highlights a missing temporal link, one which is not always measured in sensory studies and could go unrecorded, which might explain a part of plant protein-based food rejection and again pinpoints the importance of astringency as an underestimated tactile response which can not be masked by flavour or taste^9^.

To summarise, it is clear that astringency is a key tactile response ubiquitous in plant protein and we chose pea protein (PPC) as the lead candidate for revealing the mechanism. Despite being the most conspicuous as a protein in terms of astringency and concurrently negative in tactile responses, pea protein is environmentally one of most friendly plant proteins with lowest GHG emissions^60^ and nutritionally relevant^61^. This makes it a key candidate for examining its neural and cellular astringent response.

### Neural response of plant proteins

**Fig. 5:**
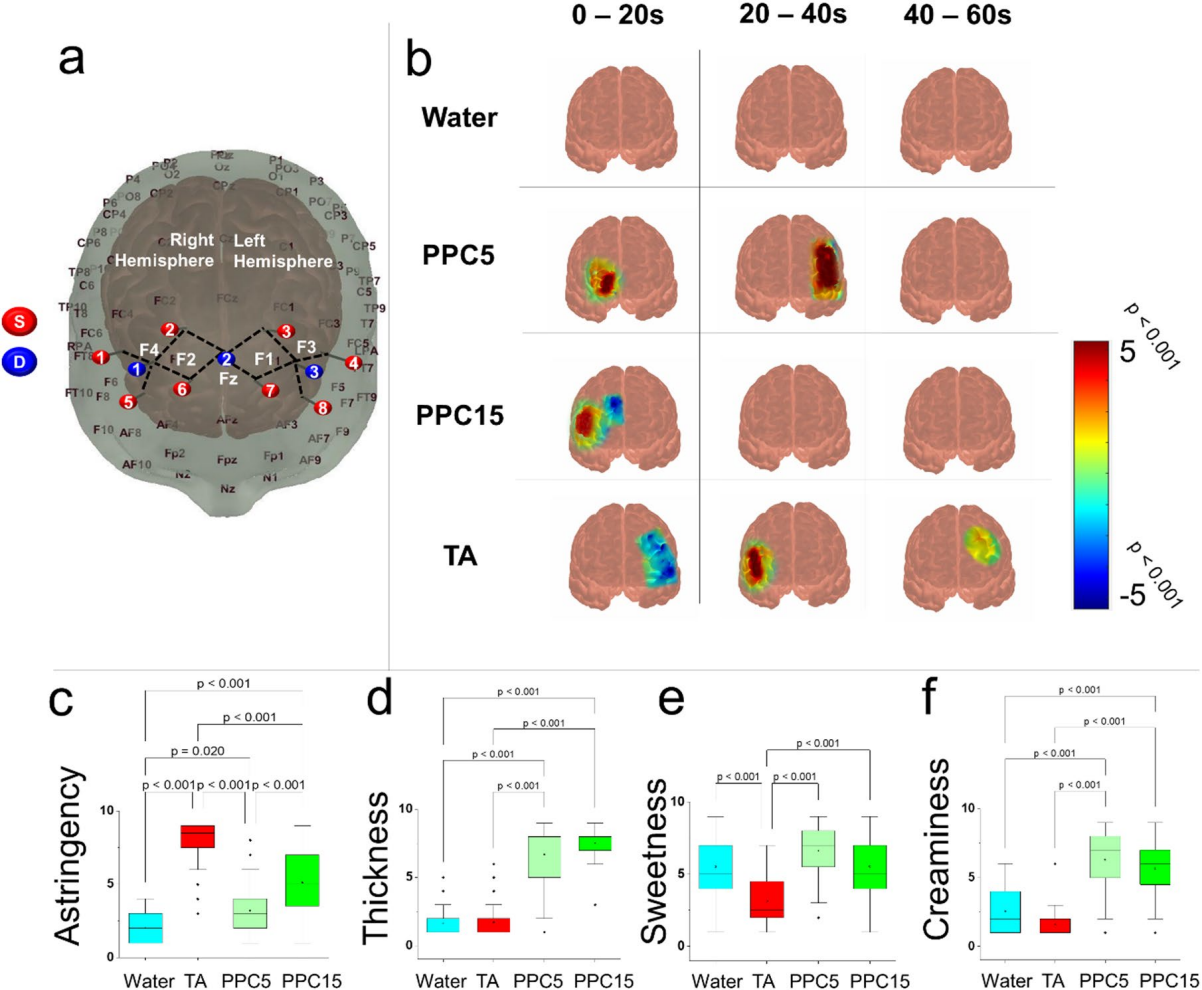
Neural response of astringent pea protein formulations compared with tannic acid (a known astringent control). Schematic diagram (**a**) of the placement of detector (blue, D1-3) and source nodes (red, S1-8) spanning the right and left dorsolateral areas (F3, F4) and dorsomedial areas (Fz) of the dorsolateral prefrontal cortex (DLPFC). (**b**) Block averaged overall neural response of changes in Oxygenated haemoglobin (HbO) using functional near-infrared spectroscopy (fNIRS) are plotted at three times points between 0–60 s. when consuming non-astringent water (Water), low astringent, viscosity matched 5 wt% total protein pea protein concentrate (PPC5), 15 wt% pea protein concentrate (PPC15) and 0.8 wt% tannic acid (TA). Beta values are plotted from T-stat (*n* = 34 participants) with the colour legend indicating magnitude of difference. A positive value (warm colours) reflects increase in HbO compared to baseline whilst a negative value (cool colours) reflects decreases in HbO. Rate-All-That-Apply (RATA) values are presented as bar and whisker plots with the interquartile range, minimum and maximum plotted for each sample in (**c**) astringency, (**d**) thickness, (**e**) sweetness and (**f**) creaminess presented as means and standard deviations of *n* = 34 with Bonferonni correction. Ethical approval was obtained from the University of Leeds (MEEC 16–046 and PSYC-475) by the Faculty Ethics Committee, University of Leeds, UK.

To elucidate the neural origin of plant protein astringency, we measured the hemodynamic changes in the dorsolateral prefrontal cortex (DLPFC) of the brain spanning the dorsolateral prefrontal areas (Brodmann area (BA) 9 and BA46 (Fig. 5a), Montreal Neurological Institute (MNI) brain locations can be found in Supplementary Table 2)). In general, the DLPFC is a higher-order functioning region of the brain responsible for thoughts, actions and emotions^62^ (refer to methods section and Supplementary Fig. 4 for protocol and Supplementary Fig. 5 for scan quality). The DLPFC area is responsible for connections in sensory processing, regulating thought and action (D1, D3). Only absolute hemodynamic response (HR) were assessed i.e. HbO increase followed by HbR decrease with recovery compared to a baseline of no stimulus, to improve accuracy of response. To our knowledge, no fNIRS study employing such scrupulous data evaluation has been conducted to examine the DLPFC in conjunction with sensory, plant protein consumption or to explore its role in understanding texture and astringency.

Tannic acid (TA) at 0.8 wt% was used as a known astringent comparator typically used as a standard in astringency studies^24,38,39^. Pea protein samples were measured with low (PPC 5 wt% protein) to high astringency (PPC15, 15 wt% protein) and water was measured as comparison where all samples were controlled for viscosity and taste (see Supplementary Fig. 6 for viscosity curves). In addition, we assessed neural activity across 60 s (Fig. 5b) in 20 s bins due to the previously found temporal nature of astringency. RATA responses were also collected (Fig. 5c-f) from the participants subjected to fNIRS tests where ratings of astringency, thickness, sweetness and creaminess were used as a measurement to relate neural and sensory data in the same participants (refer to Supplementary Fig. 7 for raw HbO, HbR and total haemoglobin (HbT) neural response across 0–60 s and Supplementary Table 3 for exact haemodynamic response values and statistics).

Firstly, consuming water revealed no significant changes compared to baseline in the neural response indicating sweetness and flavour are not coded for in the DLPFC (Fig. 5b, *p* *> 0.05*). PPC5 initiated a significant increase in HR in the right DLPFC (D1-S5, Tstat = 2.5, *p* < 0.016) at 0–20 s whilst moving to a neural response at D3-S4 and D3-S8 (Tstat = 4.0–4.2.0.2 *p* < 0.001) from 20 to 40 s (Fig. 5b). PPC15 consumption also resulted in a right DLPFC response at D1-S5 (Tstat = 4.6, *p* < 0.001) at 0–20 s and TA but at a later timepoint of 20–40 s (D1-S5, Tstat = 3.0 *p* < 0.005) (Fig. 5b). This brain area is part of BA9 of the DLPFC and since astringency is the only common denominator for these three samples (Fig. 5c-f) we suggest this region is coding for astringency.

Widespread reductions in HR were also observed for TA at 0–20 s (Fig. 5b) in left DLPFC at D3-S4 (Tstat = −2.2, *p* < 0.05) and an anterior portion of the left DLPFC in D3-S8 (Tstat =−3.4, *p* < 0.001). This is in contrast to the pea proteins which did not show an initial decrease in HR in the left hemisphere, although PPC15 did show a modest decrease in a more medial DLPFC on the right hemisphere at 0–20 s (D2-S2, Tstat = −2.4, *p* < 0.05). Worth noting is that PPC5 revealed a highly significant increase in HR in the left DLPFC at 20–40 s post consumption (D3–S4, Tstat = 4.2, *p* < 0.001; D3-S8, Tstat = 4.0, *p* < 0.001) in the same area as the TA 0–20 s response (left DLPFC), but with an opposite effect. This region has reported to be associated with pleasure from food, and this positive effect in PPC5 maybe due to it being pleasurably thick and sweet (Fig. 5d-e)^63,64^, whereas an initial rapid dislike shown by decreases in HR is not surprising for TA to which a later astringent response was found (Fig. 5b).

Strikingly, TA also induced a prolonged HR extending to 40–60 s (Fig. 5b) with activation from D3-S3 (Tstat = 2.1, *p* < 0.05), this specific area relates to left DLPFC (BA9) which is a region for memory^65^ and could be linked to storing or retrieval of information on astringency, but this needs further investigation. TA was unique in that a long neural response suggests an extensive temporal effect of astringency (Fig. 5b), as to why such response was not observed in PPC15 would likely be due to the thickness of the sample (Fig. 5d). In other words, PPC15 might have an overall lower binding to the mouth compared to TA, latter being less viscous allows for a much intense and prolonged binding effect (Supplementary Fig. 7)^66–68^.

To summarise, the right DLPFC (BA9) was shown to be stimulated by astringency in all astringent samples (Fig. 5b-c) regardless of viscosity/thickness and taste (Fig. 5d-f). The left DLPFC activation in PPC5 and TA are likely to reflect the preference in terms of liking or disliking the sample. The more astringent samples (PPC15 and TA) revealed greater and more extensive increases in NR in lateral DLPFC with TA inducing a temporal-lag and longer lasting effect. In a previous study using fMRI, tannic acid astringency has only been reported to activate primary gustatory regions of the brain inferring a possible taste association^41^. This is the first evidence to report clear responses in the right DLPFC to astringency with activation of the DLPFC would likely lead to a cascade of activation throughout the brain including the amygdala which has been reported previously^42,69^.

### Cellular response of plant protein induced astringency

**Fig. 6:**
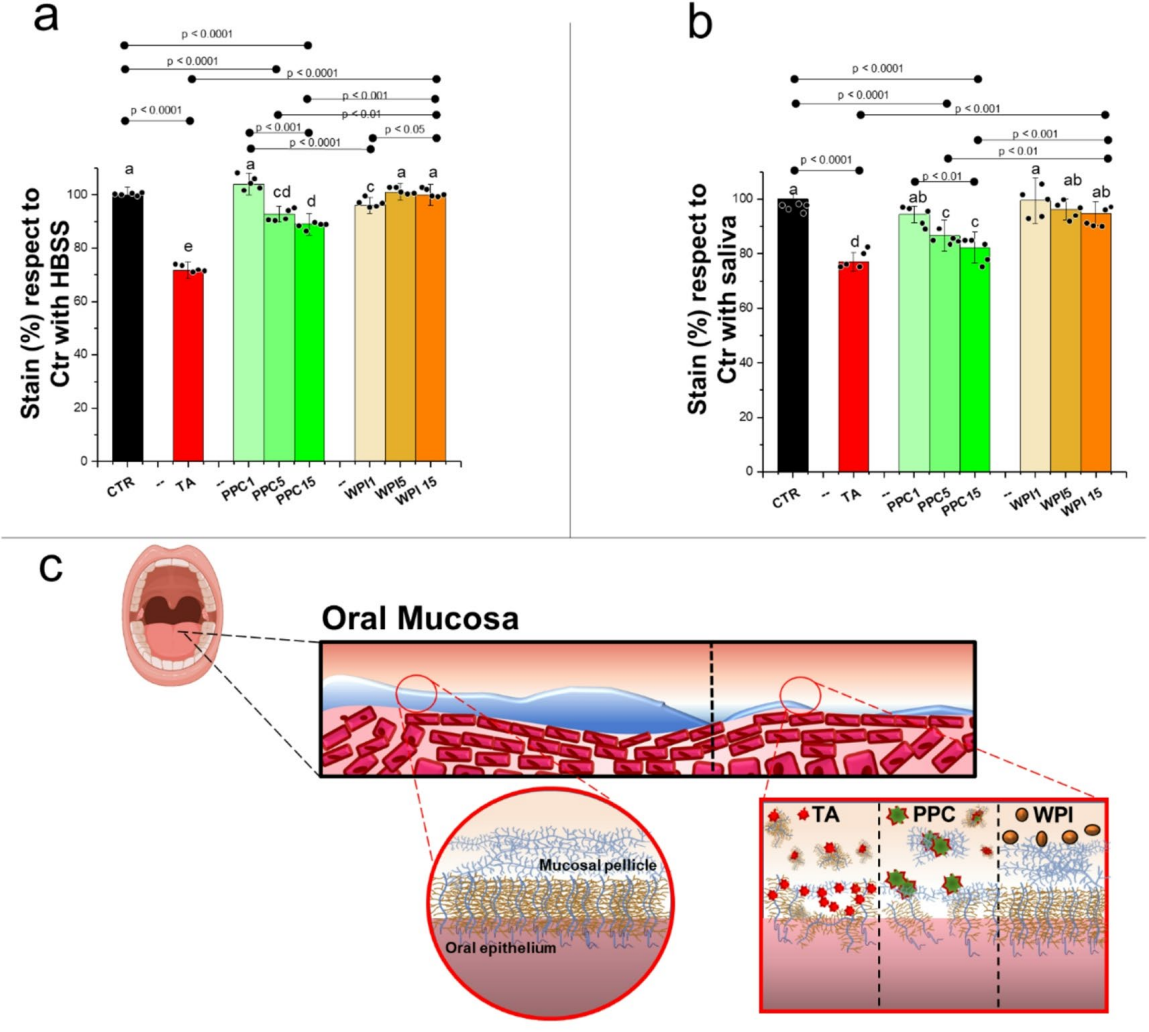
Binding capacity of plant proteins to mucosal cell lines coated with human saliva resembling tannic acid. Absorbance of Alcian blue stained (**a**) squamous cell carcinoma cells producing MUC1 (TR146-MUC1), (**b**) squamous cell carcinoma cells coated with pooled unstimulated human saliva (*n* = 10 participants) (TR146 + S) after application and washed with water (CTR), 0.8 wt% tannic acid (TA), pea protein concentrate at a total protein concentration of 1 wt% (PPC1), 5 wt% (PPC5) and 15 wt% (PPC15), and whey protein isolate at a total protein concentration of 1 wt% (WPI1), 5 wt% (WPI5) and 15 wt% (WPI15). The stain (%) is calculated in comparison to TR146-MUC1 and TR146 + S absorbance with water. Schematic representation (**c**) of TA, PPC and WPI in the oral mucosa is presented. Means and standard deviations of 5 measurements on triplicate samples (*n* = 5 × 3) are presented where statistical analysis was performed using two tailed unpaired Student’s t-test with differing lower-case letters in the same bar chart indicate a statistically significant difference (*p < 0.05*). Ethical approval was obtained from the University of Leeds (MEEC 16–046 and PSYC-475) by the Faculty Ethics Committee, University of Leeds, UK.

Having validated that indeed pea protein astringency resembles that of tannic acid in terms of neural response, next we sort to understand if binding to the salivary pellicle contributes to the astringency observed in the neural circuits. Extensive research has been conducted on TA and its protein interaction^24,70,71^ and found that TA-protein binding occurs through hydrophobic interactions between its aromatic ring structures and the hydroxyl hydrogen acceptor sites on proteins, forming stable hydrogen bonded complexes. These complexes tend to precipitate salivary proteins leading to loss of lubrication and consequently off-mouthfeel characteristics^72,73^. It is currently unknown if pea protein (PPC) instigates similar binding capabilities to TA, and if it does, it will validate the hypothesis that salivary binding triggers the mechanosensation underlying astringency^24,74^. To test this, a control of water and a well-established non-astringent protein (whey protein (WPI) 1–15 wt% total protein)^34,75,76^ at neutral pH that features a neutral taste, a pleasant texture^32^ and excellent lubrication properties was used in contrast to pea proteins^54,77–79^. Our experiments consisted of an in vitro and an ex-vivo cellular environment, one forming of TR146 cell carcinoma mucin cells expressing MUC1 (TR146-MUC1), and the second using TR146 cells inoculated with human unstimulated saliva (TR146 + S). Before exposing to protein solutions, toxicity tests were conducted to determine cell viability (Supplementary Fig. 8). The results revealed similarities in binding capabilities across both TR146-MUC1 and TR146 + S environments (Fig. 6a and b). Furthermore, TA caused the greatest difference in absorbance, thus loss of salivary and MUC1 proteins compared to the control. Specifically, TA resulted in over 25% decrease in absorbance (*p > 0.0001*), which was significantly lower than that of all other solutions (*p > 0.0001*) (Fig. 6a and b).

We also observe a significant decrease in % absorbance with 5 and 15 wt% total protein pea proteins (PPC5, PPC15) compared to control (*p < 0.05*) proving that a similar but lower binding of salivary proteins similar to that of TA can be confirmed in PPC. This degree of salivary mucin depletion correlates strongly with astringency scoring in the RATA (Fig. 2l) and neural investigation (Fig. 5c) as well as the right DLPFC activity for TA and PPC15 (Fig. 5b). Thirdly, at low concentrations (PPC1), a non-significant decrease is observed compared to water (*p > 0.05*) (Figs. 6a-b) which suggests sufficient quantities of pea protein is needed in order to aggregate and disrupt the mucinous oral film and may not induce astringency or poor tactile interaction at lower protein concentrations. Finally, upon comparing to whey protein solutions, remarkably even at concentrations of 15% a non-significant level binding to mucin occurred compared to water in both cell environments (Figs. 6a-b) proving that disruption to salivary film is induced by pea protein but not with whey protein concurrent with astringency ratings in RATA and previous study^34,75,76^. A schematic representation has been created in Fig. 6c where pre-consumption the oral mucosa is sufficiently coated in saliva, and upon consumption of TA or PPC5 or PPC15 interaction, disruption and depletion of this salivary pellicle occurs leading to astringency and related tactile consequences unlike WPI. Overall this study presents strong evidence that the binding of pea to salivary films generates an astringent response similar to those found in TA.

## Discussion

A global shift towards plant-based protein diets is urgently needed, offering both significant environmental and health benefits compared to the widespread overconsumption of animal-based proteins in current western diets. However, astringency is a prominent barrier to consistent and significant across plant protein type, with its mechanisms and sensory impact not yet fully understood, making it a major obstacle for consumer transitioning to plant-based diets. In this study, we developed a comprehensive in vitro, ex vivo and in vivo methodological approach encompassing sensory, neural and cellular characterisation that elucidates the mechanistic origin of plant protein’s astringency. Firstly, using RATA, the sensory profiles of pea, potato, and lupin proteins at total protein concentrations of 5 and 15 wt% were assessed. In particular we emphasise the perception of astringency in all protein dispersions, but also increasing upon increased protein concentration and also with time.

As well as highlighting the importance of measuring tactile attributes, over-reliance on a single plant protein source may raise the risk of protein malnutrition, particularly in developing countries where protein sources are limited and diversification is crucial. Despite the welcoming boom in plant protein-sensory research what is not frequently assessed is the sensory implications of protein mixing, thus balanced amino acid blends. We find that such mixing of proteins was associated with additional unfavourable attributes, such as graininess, astringency and roughness, warranting further investigation in future research. Additionally, we show temporal aspects of food consumption should be carefully considered in plant protein product development, as temporal studies offer a more accurate representation of typical eating experiences and highlight the subtle accumulation of sensory attributes i.e. astringency that can go overlooked and may be a key reason for consumer rejection.

Secondly, we presented neural insights of astringency using fNIRS by comparing HR of astringent with non-astringent solutions and plant proteins. We found a significant neural response on the right DLPFC in line with astringency ratings, with greater astringency showing greater dispersion and intensity of HR. FNIRS provided a complementary tool, in sensory study, offering quantitative insights that go beyond participant rating scores and accessing higher cognitive functions of memory and decision making in food choice. Finally, the mechanism behind plant protein-induced astringency was evaluated by assessing the binding properties of astringent and non-astringent solutions and proteins to saliva-inoculated TR146 cell lines and those producing MUC1. These experiments revealed that both plant proteins and well known astringent compound i.e. tannic acid prompt significant salivary binding, supporting results of both sensory perception and neural responses. This results in diminished salivary lubrication and increased oral friction, that unsurprisingly explains the coerced tactile responses of roughness, and triggers the mechanosensation associated with astringency leading to specific activity in the right DLPFC as demonstrated.

Although natural phenolics are often known to be present in in the plant proteins, the phenolic content was limited in the plant protein used in this study and this did not confound the findings. Noteworthy, we found no significant difference in total phenolic content (TPC) between WPI and PPC (Supplementary Fig. 9) in line with findings from other studies^80^. Therefore, the contribution of phenolics to plant protein astringency can be considered to be minimal. Therefore, the likely source of astringency in plant proteins is the interaction of the protein itself with saliva through hydrophobic interactions, as many plant proteins, including those tested in this study, exhibit high surface hydrophobicity^81,82^ which tend to cause protein-protein and protein-saliva interactions, as observed in the cellular scale. We therefore anticipate that physically modifying the protein structure to block or remove hydrophobic groups will reduce its binding affinity with saliva, thereby mitigating the astringency response. Such physical processing may be achieved through microgelation, forming self-assembly with polysaccharides to achieve hydration lubrication, enzymatic modification removing hydrophobic fragments^9,83–86^, which might ultimately lead to removal of salivary-binding reactive species from plant proteins. Although whey protein was introduced later as its low astringency and optimal sensory performance is widely recognised in the literature, an investigation into the neural response would be of great comparable interest and should be performed in future study. fNIRS proved to be a highly versatile neuroimaging tool that enabled advanced monitoring of neural responses during food consumption. Notably, it has demonstrated sensitivity to astringency perception. This presents a valuable method for future food research, particularly in contexts where mobility and accessibility are essential whilst consuming food and where temporal neural response is the key question. In summary, this study bridges a critical gap in sensory research on the astringency of plant proteins, which would be difficult to unravel without this integrative approach combining sensory, neural and cellular investigation. We believe such approach may unlock new insights into the intricate world of sensory science and oral processing, paving the way for fundamental understanding of complex, understudied textural challenges and might serve as a cornerstone to expedite the design of a biologically-informed sustainable food systems in the future.

## Methods

### Materials

Pea protein concentrate (PPC, Nutralys S85 XF) containing 85 wt% protein was kindly gifted by Roquette (Lestrem, France). Potato protein isolate (PoPI) was purchased from Guzmán Gastronomía (Barcelona, Spain) containing 91 wt% soluble protein^54^. Whey protein isolate (WPI) was kindly gifted by Fonterra (Palmerston North, New Zealand) containing at least 95 wt% protein. Lupin protein isolate (LPI) was purchased from Prolupin (GmbH, Grimmen, Germany) containing 90 wt% protein. Food grade tannic acid (TA) was purchased from APC pure (UK) and xanthan gum purchased from Special ingredients (UK). Madagascan vanilla extract and sucralose sweetener (maltodextrin, sucralose (1%)) were purchased from a local supermarket (Morrisons, UK).

For the cell culture, the squamous cell carcinoma cell line (TR146, wild type) was kindly provided INRAE (National Research Institute for Agriculture, Food and the Environment) by Prof F.M. Goycoolea at the University of Murcia, Spain, and mucin-expressing TR146 cell line (TR146/MUC1) was gifted by Dr F. Canon (INRAE, France)^87^. Reagents for cell culture were purchased from Gibco ThermoFisher Scientific (Loughborough, UK), including Dulbecco’s Modified Eagle Medium (DMEM), Dulbecco’s Phosphate-Buffered Saline (DPBS), Hanks’ balanced salt solution (HBSS), penicillin/streptomycin (P/S), and trypsin/Ethylenediaminetetraacetic (EDTA) whereas foetal bovine serum (FBS) was obtained from Sigma-Aldrich, Dorset, UK. Alcian blue stain was purchased from Epredia (Runcorn, UK), and neutral red dye was purchased from Sigma-Aldrich, Dorset, UK. The glacial acetic acid and ethanol were obtained from ThermoFisher, UK and dimethyl sulfoxide (DMSO) were obtained from Santa Cruz Biotechnology (Heidelberg, Germany). Milli-Q water was purified using Milli-Q apparatus, Millipore Corp., Bedford, MA, USA.

### Participants

Healthy male and female volunteers (*n* = 135 for both RATA sensory and fNIRS neural study) between 18 and 55 years old were recruited using advertisement of posters to students and staff at the University of Leeds, UK and informed consent was obtained from the participants. Participants were excluded if they were underweight (BMI < 18.5 kg/m^2^), overweight or obese (BMI ≥ 25 kg/m^2^), were smokers, had suffered from long-term effects of COVID-19, had oral problems or were using medication that would affect ability to eat/sense/digest food, were pregnant/lactating, had a food allergy/intolerance and free of neurological and psychiatric disorders. Participants were informed not to eat, and only drink water 2 h before the sensory procedure. Participants were informed on the purpose and safety of experiments with written consent forms collected before participation and received financial reimbursement for compensation after study. Ethical approval was obtained from the University of Leeds (MEEC 16–046 and PSYC-475) by the Faculty Ethics Committee, University of Leeds, UK and was conducted in accordance with the Declaration of Helsinki and the guidelines for human research.

### Rate-All-That-Apply (RATA) procedure

Before RATA, a preliminary check all that apply (CATA) test was performed for all solutions to determine taste, texture and afterfeel (AF) attributes for the RATA investigation. A total of 100 volunteers were recruited, 40 male and 60 female between age of 18–40 with a mean age of 24 ± 4.2 years. Participants were screened beforehand using an online health questionnaire and each participant came in once. The proteins in relevant concentrations were prepared in distilled water and flavoured using 1.2 wt% vanilla extract and 1 wt% sucralose sweeter (0.01 wt% total sucralose in maltodextrin). Exactly 20 mL of each sample was provided in black opaque cups with a lid and straw to prevent appearance discrepancies affecting the scoring. The samples were randomly labelled with a three-digit code and provided one at a time in random order generated using compusense (v5.0, Ontario, Canada). Prior to attending, participants were familiarised with definitions and examples of the chosen 17 RATA sensory attributes divided over three categories: Taste (5), texture (9) and afterfeel (3). Definitions were also explained before commencing the session and provided throughout the assessment (Supplementary Table 1). Participants rated the attributes on a 1–9 scale (anchored low to high) and were allowed multiple tastings. After feel scores were assessed 10 s after final consumption of sample. A 3 min washout period between samples was provided with crackers and water to wash out and neutralise the palate between the samples.

### fNIRS

Oxyhaemoglobin (HbO) and deoxyhaemoglobin (HbR) blood (µmol/L) were measured using OxyMon MkII functional near-infared spectroscopy (fNIRS) system (Artinis Medical Systems, B.V., The Netherlands). To monitor such changes, we utilised infrared light of 765 nm and 855 nm wavelengths relating to the absorption spectrum of haemoglobin (700–900 nm). Using this wavelength of light, we detect the differential absorption of HbO and HbR reflecting increase in arteriolar vasodilation and cerebral blood flow corresponding to prefrontal cortex activation. Differential path length factor (DPF) was calculated from participants’ age using formula: DPF = 4.99 + 0.067*(age^0.814). Detector optodes were embedded onto a black cap secured with elastic to optimise signal to noise ratio by removing ambient light. Placement of the cap was done by identifying fiducial points of the naison, inion and left/right preauricular joints. A drip stand was used to suspend cables to reduce artefacts associated with likelihood of movement. 3 × detector optodes (Blue dots D1-3 Fig. 4.4a) and 8 source optodes (Yellow dots S1-8 Fig. 4.4a). This optode configuration resulted in 12 channels (8 split, 4 unsplit) sampling at a rate of 10 Hz. This arrangement spanned the left, midline and right prefrontal cortex region of the brain with D1 and D3 corresponding to the 10–20 EEG standardised MNI coordinate system F4 (right dorsolateral prefrontal cortex, DLPFC), F3 (left DLPFC) (see Supplementary Table 2 for estimates of MNI coordinates and brain location for each of the 12 channels using AtlasViewer^88^. Each of the 12 channels were 3.5 cm long with a recording depth of 2–3 mm from the surface of the cortex with sensitivity analysis of optodes showing good sensitivity across the prefrontal cortex (see Supplementary Fig. 5).

A total of 35 healthy volunteers were recruited, 14 male and 21 female between age of 18 and 55 with a mean age of 23.3 ± 3.9 years. A quality check was performed using QTNirs in MatLab (https://github.com/lpollonini/qt-nirs), and channels that did not reach a 70% threshold of signal quality were removed/trimmed from further analysis. In addition, 1 participant was excluded due to poor signal quality across the entire session. Brain responses during the consumption of four samples were collected. Viscosity was standardised between control non-astringent and astringent samples (Supplementary Fig. 6) with xanthan gum added to PPC5 to exclude viscosity-associated brain response effects. Participants sat on a chair facing a white featureless wall in front of laptop, the room was dimly lit to reduce any outside distraction resulting in noise. The study procedure was created using Gorilla™ (www.gorilla.sc) where the participant filled out an information sheet, consent form and were provided with written and verbal instructions released at specific timings outlined below. The fNIRS cap with optodes was fitted on the scalp and participants were asked to limit unnecessary movement and to complete tasks in silence to reduce data artifacts. Participants firstly had 40 s to consume a small cracker and water, then a 60 s baseline waiting period commenced followed by 40 s to consume 20 mL of one of the 4 samples provided in a randomized order to each participant (Supplementary Fig. 4). Following this the participants then rated sample texture and taste attributes on a laptop, and these steps were repeated three more times to obtain brain response to all the four samples (PPC5, PPC15, TA and water). Experimenters placed markers during data collection at the start of each new sample allowing temporal realignment of baseline and tasting phases with fNIRS responses for the analysis stage. Astringency, roughness, creaminess, sweetness and thickness texture attributes were rated similarly to RATA procedure described previously whereby definitions were familiarised before starting procedure.

Data pre-processing and analysis was done using the NIRS Brain Analyzer Toolbox (operating in Matlab (MatLab 2023a, Mathworks), for pipeline of data processing see Supplementary Fig. 4. Files were stored using the Brain Imaging Data Structure and converted into the universal *.snirf format. Pre-processing was performed to remove biological and technical artefacts converting haemodynamic intensity raw data into optical density applying the following protocol: a PCA filter was used to remove first principal components of noise, motion artefacts were removed using an auxiliary high pass filter (HPF, −0.020 Hz) and low pass filter (LPF, 1.00 Hz) and converted to optical density. Finally, the modified Beer-Lambert law (MBLL) was applied to obtain HbO and HbR concentrations. We used a General Linear Model utilising a finite impulse response (FIR) to model the data and generate block averages for each of the 12 channels. Data was split into 0–20 s, 20–40 s and 40–60 s post sample ingestion to summarise changes in brain activity to tasting, and more prolonged after-effects respectively. Individual subject data was then subjected to a mixed effects group ANOVA resulting in a Δ HbO for each sample (for data pipeline refer to Supplementary Fig. 4). Only data that reached a *p* < 0.05 significance level alongside showing contrasting HbR changes evidencing neurovascular coupling are reported (Supplementary Figs. 4 and 5) and subsequently mapped onto a template brain using AtlasViewer (Fig. 5a in manuscript). The heatmaps show T-stats of increases (warm colours) and decreases (cool colours) in HR compared to baseline (Fig. 5b).

### Cell culture and maintenance

TR146-MUC1 and TR146-S cells were cultivated in Dulbecco’s Modified Eagle Medium (DMEM) (4.5 g L^−1^ *D*-glucose with pyruvate) supplemented with 10% Fetal Bovine Serum and 1% Penicillin-Streptomycin (P/S) under standard conditions (5% CO_2_, 37 °C) with medium replacement every other day. After reaching 80–90% confluence, the cells were rinsed with DPBS (1×) and detached with trypsin/EDTA (1×). Cells were used for experiments up to 15 inhouse passages.

### Saliva collection

Unstimulated saliva was collected from pooled healthy non-smoker participants (*n* = 10, 6 female, 4 male aged between 23 and 31) who were refrained from eating/drinking for at least 2 h before saliva collection. The volunteers spat saliva into a collection tube allowing collection for 10 min under unstimulated conditions. Immediately after collection, the saliva was pooled and centrifuged at 4,000 *g* for 5 min. The supernatant was applied to the TR146 cells. Ethics approved from the University of Leeds (MEEC 16–046 ethics approved by the Faculty Ethics Committee, University of Leeds).

### Cytotoxicity assay

Toxicity of PPC, WPI and TA towards the cells was determined by neutral red assay, which is based on the lysosomal accumulation of dye in viable cells (Supplementary Fig. 8). To this end, cells were seeded in 24-well plates at a density of 8 × 10^4^ cells cm^−2^. Upon reaching 95% confluence, the medium was replaced by PPC or WPI (10–150 mg mL^−1^), and TA (2–8 mg mL^−1^) dissolved in medium and incubated for 1–24 h, meanwhile the medium control and positive control (5% DMSO), *v/v*) were performed in parallel to each replicate. Cytotoxicity of pooled unstimulated human saliva (1–400 µL mL^−1^) towards TR146 cells (wild type) was also examined at 1 h duration. After incubation, the treatments were removed and replaced by the FBS-free medium containing neutral red (40 µg mL^−1^) followed by 3 h incubation. After washing with DPBS, the cells were destained in water/ethanol/glacial acid (49:50:1, *v/v/v*) and shaken horizontally for 5 min in the dark. Absorbance readings were recorded on a Tecan Spark 10 M™ at 540 nm. The relative cell viability was calculated in percentage of the medium control.

### Alcian blue staining

Briefly, this methodology determined how much mucin binds with the solutions we expose to the ex vivo environments, therefore removed compared to a control with just the continuous phase. The amount of mucin bound to the epithelial cell layer was estimated by the alcian blue method when alcian blue interacts with the anionic carboxylate and sulphate groups of mucin. The two ex vivo environments were namely, squamous cell carcinoma cells producing MUC1 (TR146-MUC1) and squamous cell carcinoma cells coated with pooled unstimulated human saliva (TR146 + S). For the first environment TR146-MUC1 cells, grown in 24-well plates to 95% confluence were equilibrated in HBSS for 1 h before adding 1 wt% − 15 wt% (10–150 mg mL^−1^) concentrations of PPC or WPI or 0.2 wt% − 0.8 wt% (2–8 mg mL^−1^) TA dissolved in HBSS. The duration of treatment was determined to be 1 h on account of the cell viability (Supplementary Fig. 8) and oral administration conditions. For the second environment TR146 wild type cells were incubated in 30% pooled, unstimulated saliva in HBSS solution (*v/v*) for 1 h prior to the treatments of proteins and TA. For both environments after incubation with respective solutions with additions (i.e. PPC, WPI or TA) were removed, cells were washed twice with DPBS, and 1% alcian blue dye (*w/v*) in 3% acetic acid (*v/v*) was subsequently applied on the cell surface followed by 15 min incubation at room temperature in the dark. After removing the dye, cells were rinsed in DPBS for 3–4 times prior to the destaining procedure in DMSO. The plate was shaken horizontally for 5 min under light shielding, then absorbance was measured at 675 nm using Tecan Spark reader. The proportion of mucin was calculated in percentage respect to the control with saliva or with HBSS that contained no PPC, WPI or TA.

### Total phenolic content determination

The total phenolic content (TPC) of WPI and PPC extracts was determined as gallic acid equivalent (GAE) using the Folin-Ciocalteu reagent^89^. Briefly, 0.1 g of WPI and PPC powder was mixed with 1 ml of 80% methanol containing 0.1% hydrochloric acid and incubated in a shaking stirrer bath at 40 °C for 1 h. The solution was then centrifuged at 4000 RPM for 20 min, and the supernatant was collected. An additional 1 ml of methanol-HCl solution was added to the remaining pellet, and the extraction procedure was repeated. The pooled supernatants were adjusted to a final volume of 3 ml with methanol-HCl and stored at −80 °C until use. For TPC measurement, 15 µl of the protein extract was mixed with 200 µl of Milli-Q water, 20 µl of 7.0% sodium carbonate, and 15 µl of Folin-Ciocalteu reagent in a 96-well plate. The mixture was incubated at room temperature for 60 min, and absorbance was measured at 765 nm using a Tecan Spark 10 M™ spectrophotometer.

### Statistical analysis

Unless specified else, all results are reported as means and standard deviations of at least three repeats in three independent experiments. Statistical analysis was performed using R (Version 4.4.2) and OriginPro2022 (OriginLab Corporation, Northampton, MA, USA) where the significance between data sets was calculated using one-way analysis of variance (ANOVA) with Tukey post hoc test, with significance level threshold at *p < 0.05.* For sensory evaluation results, Bonferroni correction was applied to account for multiple comparisons, and data were normalised prior to statistical testing to ensure comparability. PCA was employed as an initial step in multivariate analysis for dimensionality reduction. PCA was used to calculate principal components, which summarise the variance in the data by reducing the contribution of less significant variables. The percentage variance explained by each principal component is reported alongside biplots, which illustrate the relationships between samples (scores) and factors (loadings) aiding in identifying patterns and correlations within the dataset.

## Supplementary Information

Below is the link to the electronic supplementary material.

Supplementary Material 1

## Data Availability

All data generated or analysed during this study are included in this published article and its Supplementary Information files. The raw data of this article are available in the University of Leeds database repository https://doi.org/10.5518/1710.

## References

[1] Xu, X. et al. Global greenhouse gas emissions from animal-based foods are twice those of plant-based foods. *Nat. Food*. **2**, 724–732 (2021). 37117472 10.1038/s43016-021-00358-x

[2] Kennedy, J., Alexander, P., Taillie, L. S. & Jaacks, L. M. Estimated effects of reductions in processed meat consumption and unprocessed red meat consumption on occurrences of type 2 diabetes, cardiovascular disease, colorectal cancer, and mortality in the USA: a microsimulation study. *Lancet Planet. Health*. **8**, e441–e451 (2024). 38969472 10.1016/S2542-5196(24)00118-9

[3] Papier, K. et al. Meat consumption and risk of 25 common conditions: outcome-wide analyses in 475,000 men and women in the UK biobank study. *BMC Med.* **19**, 53 (2021). 33648505 10.1186/s12916-021-01922-9 PMC7923515

[4] Cheynier, V. Phenolic compounds: from plants to foods. *Phytochem. Rev.* **11**, 153–177 (2012).

[5] Brown, F. N., Mackie, A. R., He, Q., Branch, A. & Sarkar, A. Protein–saliva interactions: a systematic review. *Food Funct.* **12**, 3324–3351 (2021). 33900320 10.1039/d0fo03180a

[6] Tanger, C. et al. Influence of pea and potato protein microparticles on texture and sensory properties in a Fat-Reduced model milk dessert. *ACS Food Sci. Technol.* **2**, 169–179 (2022).

[7] Vlădescu, S-C. et al. Protein-induced delubrication: how plant-based and dairy proteins affect mouthfeel. *Food Hydrocoll.* **134**, 107975 (2023).

[8] Sarkar, A. Oral astringency in plant proteins: an underestimated issue in formulating Next-Generation plant-Based foods. *Annu Rev. Food Sci. Technol* **15**, (2024). 10.1146/annurev-food-072023-034510 38316152

[9] Sarkar, A. Oral astringency in plant proteins: an underestimated issue in formulating next-generation plant-based foods. *Annual Rev. Food Sci. Technol.* **15**, 103–123 (2024). 38316152 10.1146/annurev-food-072023-034510

[10] Kim, M., Heo, G. & Kim, S-Y. Neural signalling of gut mechanosensation in ingestive and digestive processes. *Nat. Rev. Neurosci.* **23**, 135–156 (2022). 34983992 10.1038/s41583-021-00544-7

[11] Testing & ASf Materials. Annual Book of ASTM Standards: Nonferrous Metal Products. Die-cast Metals; Aluminum and Magnesium Alloys. (ed^(eds). Astm (1983).

[12] Rossetti, D., Bongaerts, J. H. H., Wantling, E., Stokes, J. R. & Williamson, A. M. Astringency of tea catechins: more than an oral lubrication tactile percept. *Food Hydrocoll.* **23**, 1984–1992 (2009).

[13] Silletti, E., Vingerhoeds, M. H., Norde, W. & van Aken, G. A. Complex formation in mixtures of lysozyme-stabilized emulsions and human saliva. *J. Colloid Interface Sci.* **313**, 485–493 (2007). 17574261 10.1016/j.jcis.2007.05.030

[14] Vardhanabhuti, B., Kelly, M. A., Luck, P. J., Drake, M. A. & Foegeding, E. A. Roles of charge interactions on astringency of Whey proteins at low pH. *J. Dairy Sci.* **93**, 1890–1899 (2010). 20412902 10.3168/jds.2009-2780

[15] Sarkar, A., Goh, K. K. T. & Singh, H. Colloidal stability and interactions of milk-protein-stabilized emulsions in an artificial saliva. *Food Hydrocoll.* **23**, 1270–1278 (2009).

[16] Luck, P., Vårum, K. M. & Foegeding, E. A. Charge related astringency of chitosans. *Food Hydrocoll.* **48**, 174–178 (2015).

[17] Lim, J. & Lawless, H. T. Oral sensations from iron and copper sulfate. *Physiol. Behav.* **85**, 308–313 (2005). 15935409 10.1016/j.physbeh.2005.04.018

[18] Soares, S. et al. Reactivity of human salivary proteins families toward food polyphenols. *J. Agric. Food Chem.* **59**, 5535–5547 (2011). 21417408 10.1021/jf104975d

[19] Canon, F. et al. Binding site of different tannins on a human salivary proline-rich protein evidenced by dissociative photoionization tandem mass spectrometry. *Tetrahedron* **71**, 3039–3044 (2015).

[20] Ma, S., Lee, H., Liang, Y. & Zhou, F. Astringent mouthfeel as a consequence of lubrication failure. *Angew. Chem. Int. Ed.* **55**, 5793–5797 (2016). 10.1002/anie.201601667 27059282

[21] Nayak, A. & Carpenter, G. H. A physiological model of tea-induced astringency. *Physiol. Behav.* **95**, 290–294 (2008). 18590751 10.1016/j.physbeh.2008.05.023

[22] Biegler, M., Delius, J., Käsdorf, B. T., Hofmann, T. & Lieleg, O. Cationic astringents alter the tribological and rheological properties of human saliva and salivary mucin solutions. *Biotribology* **6**, 12–20 (2016).

[23] Canon, F. et al. Perspectives on astringency sensation: an alternative hypothesis on the molecular origin of astringency. *J. Agric. Food Chem.* **69**, 3822–3826 (2021). 33682421 10.1021/acs.jafc.0c07474

[24] Schöbel, N. et al. Astringency is a trigeminal sensation that involves the activation of G Protein–Coupled signaling by phenolic compounds. *Chem. Senses*. **39**, 471–487 (2014). 24718416 10.1093/chemse/bju014

[25] Sarkar, A. & Dickinson, E. Sustainable food-grade Pickering emulsions stabilized by plant-based particles. *Curr. Opin. Colloid Interface Sci.* **49**, 69–81 (2020).

[26] García Arteaga, V. et al. Screening of twelve pea (Pisum sativum L.) cultivars and their isolates focusing on the protein Characterization, Functionality, and sensory profiles. *Foods* **10**, 758 (2021). 33918162 10.3390/foods10040758 PMC8065828

[27] Cosson, A., Souchon, I., Richard, J., Descamps, N. & Saint-Eve, A. Using multiple sensory profiling methods to gain insight into Temporal perceptions of pea Protein-Based formulated foods. *Foods* **9**, 969 (2020). 32707881 10.3390/foods9080969 PMC7466195

[28] Liu, Y., Toro-Gipson, R. S. D. & Drake, M. Sensory properties and consumer acceptance of ready-to-drink vanilla protein beverages. *J. Sens. Stud.* **36**, e12704 (2021).

[29] Zheng, Y. X. et al. Amyloid fibrils-regulated high-moisture extruded soy proteins: Texture, structure, and taste. *Food Hydrocolloids* **144**, 109026 (2023).

[30] Stribiţcaia, E., Evans, C. E. L., Gibbons, C., Blundell, J. & Sarkar, A. Food texture influences on satiety: systematic review and meta-analysis. *Sci. Rep.* **10**, 12929 (2020). 32737349 10.1038/s41598-020-69504-y PMC7395742

[31] Paul, V., Tripathi, A. D., Agarwal, A., Kumar, P. & Rai, D. C. Tribology – Novel oral processing tool for sensory evaluation of food. *LWT* **160**, 113270 (2022).

[32] Kew, B., Holmes, M., Stieger, M. & Sarkar, A. Review on fat replacement using protein-based microparticulated powders or microgels: A textural perspective. *Trends Food Sci. Technol.* **106**, 457–468 (2020). 33380775 10.1016/j.tifs.2020.10.032 PMC7763486

[33] Kew, B. et al. Relating tribology to astringency perception in acidic plant protein-fortified fiber-based smoothies. *Food Hydrocoll.* **171**, 111770 (2026).

[34] Ye, A., Streicher, C. & Singh, H. Interactions between Whey proteins and salivary proteins as related to astringency of Whey protein beverages at low pH. *J. Dairy Sci.* **94**, 5842–5850 (2011). 22118074 10.3168/jds.2011-4566

[35] De Araujo, I. E. & Rolls, E. T. Representation in the human brain of food texture and oral fat. *J. Neurosci.* **24**, 3086–3093 (2004). 15044548 10.1523/JNEUROSCI.0130-04.2004 PMC6729847

[36] De Marcina, B. et al. PA. Functional magnetic resonance imaging assessment of the cortical representation of oral viscosity. *Journal of Texture Studies* **38**, 725–737 (2007).

[37] McCabe, C. & Rolls, E. T. Umami: a delicious flavor formed by convergence of taste and olfactory pathways in the human brain. *The European Journal of Neuroscience* **25**, 1855–1864 (2007). 10.1111/j.1460-9568.2007.05445.x 17432971

[38] Zhu, Y., Thaploo, D., Han, P. & Hummel, T. Processing of Sweet, astringent and pungent oral stimuli in the human brain. *Neuroscience* **520**, 144–155 (2023). 36966878 10.1016/j.neuroscience.2023.03.011

[39] Kishi, M., Sadachi, H., Nakamura, J. & Tonoike, M. Functional magnetic resonance imaging investigation of brain regions associated with astringency. *Neurosci. Res.* **122**, 9–16 (2017). 28366831 10.1016/j.neures.2017.03.009

[40] Glover, G. H. Overview of functional magnetic resonance imaging. *Neurosurg. Clin. N. Am.* **22**, 133–139 (2014) (**vii**). 10.1016/j.nec.2010.11.001 PMC3073717 21435566

[41] Iannilli, E., Noennig, N., Hummel, T. & Schoenfeld, A. M. Spatio-temporal correlates of taste processing in the human primary gustatory cortex. *Neuroscience* **273**, 92–99 (2014). 24846613 10.1016/j.neuroscience.2014.05.017

[42] Rolls, E. T. Taste, olfactory, and food reward value processing in the brain. *Prog Neurobiol.* **127–128**, 64–90 (2015). 25812933 10.1016/j.pneurobio.2015.03.002

[43] Laves, K., Mehlhose, C. & Risius, A. Sensory measurements of taste: aiming to visualize sensory differences in taste perception by Consumers—An experiential fNIRS approach. *Journal Int. Food & Agribusiness Marketing*, **35**(5), 1–21 (2022).

[44] Soares, S. et al. Development of a new cell-based oral model to study the interaction of oral constituents with food polyphenols. *J. Agric. Food Chem.* **67**, 12833–12843 (2019). 31657214 10.1021/acs.jafc.9b05575

[45] Soares, S. et al. Oral interactions between a green tea Flavanol extract and red wine anthocyanin extract using a new cell-based model: insights on the effect of different oral epithelia. *Sci. Rep.* **10**, 12638 (2020). 32724226 10.1038/s41598-020-69531-9 PMC7387539

[46] Tulbek, M. C., Lam, R. S. H., Wang, Y., Asavajaru, P. & Lam, A. Chapter 9 - Pea: A Sustainable Vegetable Protein Crop. In: *Sustainable Protein Sources* (ed^(eds Nadathur SR, Wanasundara JPD, Scanlin L). Academic Press (2017).

[47] Lucas, M. M. et al. The future of lupin as a protein crop in Europe. *Frontiers in Plant Science* **6**, (2015). 10.3389/fpls.2015.00705 PMC4561814 26442020

[48] Sim, S. Y. J., SRV, A., Chiang, J. H. & Henry, C. J. Plant Proteins for Future Foods: A Roadmap. 10, 1967 (2021). 10.3390/foods10081967 PMC8391319 34441744

[49] Schmidt, J. M. et al. Gel properties of potato protein and the isolated fractions of patatins and protease inhibitors – Impact of drying method, protein concentration, pH and ionic strength. *Food Hydrocoll.* **96**, 246–258 (2019).

[50] Ares, G. et al. Evaluation of a rating-based variant of check-all-that-apply questions: Rate-all-that-apply (RATA). *Food Qual. Prefer.* **36**, 87–95 (2014).

[51] Guinard, J-X., Pangborn, R. M. & Lewis, M. J. The Time-Course of astringency in wine upon repeated ingestion. *American Journal of Enology and Viticulture* **37**, 184–189 (1986).

[52] Cosson, A., Souchon, I., Richard, J., Descamps, N. & Saint-Eve, A. Using multiple sensory profiling methods to gain insight into Temporal perceptions of pea protein-based formulated foods. *Foods* **9**, 969 (2020). 10.3390/foods9080969 PMC7466195 32707881

[53] Sarkar, A. & Krop, E. M. Marrying oral tribology to sensory perception: a systematic review. *Curr. Opin. Food Sci.* **27**, 64–73 (2019). 31903320 10.1016/j.cofs.2019.05.007 PMC6936954

[54] Kew, B., Holmes, M., Stieger, M. & Sarkar, A. Oral tribology, adsorption and rheology of alternative food proteins. *Food Hydrocoll.* **116**, 106636 (2021).

[55] Zembyla, M. et al. Surface adsorption and lubrication properties of plant and dairy proteins: A comparative study. *Food Hydrocoll.* **111**, 106364 (2021). 33536697 10.1016/j.foodhyd.2020.106364 PMC7607376

[56] Pownall, T. L., Udenigwe, C. C. & Aluko, R. E. Amino acid composition and antioxidant properties of pea seed (Pisum sativum L.) enzymatic protein hydrolysate fractions. *J. Agric. Food Chem.* **58**, 4712–4718 (2010). 20359226 10.1021/jf904456r

[57] Young, V. R. & Pellett, P. L. Plant proteins in relation to human protein and amino acid nutrition. *Am. J. Clin. Nutr.* **59**, 1203s–1212s (1994). 8172124 10.1093/ajcn/59.5.1203S

[58] Galili, G. & Amir, R. Fortifying plants with the essential amino acids lysine and methionine to improve nutritional quality. *Plant. Biotechnol. J.* **11**, 211–222 (2013). 23279001 10.1111/pbi.12025

[59] Giacalone, D., Clausen, M. P. & Jaeger, S. R. Understanding barriers to consumption of plant-based foods and beverages: insights from sensory and consumer science. *Curr. Opin. Food Sci.* **48**, 100919 (2022).

[60] Poore, J. & Nemecek, T. Reducing food’s environmental impacts through producers and consumers. *Science* **360**, 987–992 (2018). 29853680 10.1126/science.aaq0216

[61] Shanthakumar, P. et al. The current situation of pea protein and its application in the food industry. *Molecules* **27**, 5354 (2022). 10.3390/molecules27165354 PMC9412838 36014591

[62] Wood, J. N. & Grafman, J. Human prefrontal cortex: processing and representational perspectives. *Nat. Rev. Neurosci.* **4**, 139–147 (2003). 12563285 10.1038/nrn1033

[63] Liu, J., Cattaneo, C., Papavasileiou, M., Methven, L. & Bredie, W. L. P. A review on oral tactile sensitivity: measurement techniques, influencing factors and its relation to food perception and preference. *Food Qual. Prefer.* **100**, 104624 (2022).

[64] Drewnowski, A. & Greenwood, M. R. C. Cream and sugar: human preferences for high-fat foods. *Physiol. Behav.* **30**, 629–633 (1983). 6878464 10.1016/0031-9384(83)90232-9

[65] KluneCB, Jin, B. & DeNardoLA Linking mPFC circuit maturation to the developmental regulation of emotional memory and cognitive flexibility. *eLife* **10**, e64567 (2021). 33949949 10.7554/eLife.64567 PMC8099425

[66] Courregelongue, S., Schlich, P. & Noble, A. C. Using repeated ingestion to determine the effect of sweetness, viscosity and oiliness on Temporal perception of soymilk astringency. *Food Qual. Prefer.* **10**, 273–279 (1999).

[67] Peleg, H. & Noble, A. C. Effect of viscosity, temperature and pH on astringency in cranberry juice. *Food Qual. Prefer.* **10**, 343–347 (1999).

[68] Smith, A. K., June, H. & Noble, A. C. Effects of viscosity on the bitterness and astringency of grape seed tannin. *Food Qual. Prefer.* **7**, 161–166 (1996).

[69] Rolls, E. T. The texture and taste of food in the brain. *J. Texture Stud.* **51**, 23–44 (2020). 31598975 10.1111/jtxs.12488

[70] Payne, C., Bowyer, P. K., Herderich, M. & Bastian, S. E. P. Interaction of astringent grape seed procyanidins with oral epithelial cells. *Food Chem.* **115**, 551–557 (2009).

[71] Kurogi, M. et al. Auto-oxidation products of Epigallocatechin gallate activate TRPA1 and TRPV1 in sensory neurons. *Chem. Senses*. **40**, 27–46 (2014). 25422365 10.1093/chemse/bju057

[72] Charlton, A. J. et al. Polyphenol/Peptide binding and precipitation. *J. Agric. Food Chem.* **50**, 1593–1601 (2002). 11879042 10.1021/jf010897z

[73] Frazier, R. A., Papadopoulou, A., Mueller-Harvey, I., Kissoon, D. & Green, R. J. Probing Protein – Tannin interactions by isothermal Titration microcalorimetry. *J. Agric. Food Chem.* **51**, 5189–5195 (2003). 12926857 10.1021/jf021179v

[74] Shuanhong Ma, H. L. & Liang, Y. Feng Zhou. Astringent mouthfeel as a consequence of lubrication failure. *Angewandte Chemie Int.* **55**, 5793–5797 (2016). 10.1002/anie.201601667 27059282

[75] Beecher, J. W., Drake, M. A., Luck, P. J. & Foegeding, E. A. Factors regulating astringency of Whey protein beverages. *J. Dairy Sci.* **91**, 2553–2560 (2008). 18565912 10.3168/jds.2008-1083

[76] Carter, B. G. & Drake, M. Influence of oral movement, particle size, and zeta potential on astringency of Whey protein. *J. Sens. Stud.* **36**, e12652 (2021).

[77] Sánchez-Obando, J-D., Cabrera-Trujillo, M. A., Olivares-Tenorio, M-L. & Klotz, B. Use of optimized microparticulated Whey protein in the process of reduced-fat spread and petit-suisse cheeses. *LWT* **120**, 108933 (2020).

[78] Sarkar, A., Kanti, F., Gulotta, A., Murray, B. S. & Zhang, S. Aqueous lubrication, structure and rheological properties of Whey protein microgel particles. *Langmuir* **33**, 14699–14708 (2017). 29193975 10.1021/acs.langmuir.7b03627

[79] Campbell, C. L., Foegeding, E. A. & van de Velde, F. A comparison of the lubrication behavior of Whey protein model foods using tribology in linear and elliptical movement. *J. Texture Stud.* **48**, 335–341 (2017). 28556911 10.1111/jtxs.12278

[80] Sawicki, T., Jabłońska, M., Danielewicz, A. & Przybyłowicz, K. E. Phenolic compounds profile and antioxidant capacity of plant-based protein supplements. *Molecules* **29**, 2101 (2024). 38731592 10.3390/molecules29092101 PMC11085232

[81] Grossmann, L. & McClements, D. J. Current insights into protein solubility: A review of its importance for alternative proteins. *Food Hydrocoll.* **137**, 108416 (2023).

[82] McLauchlan, J., Tyler, A. I. I., Chakrabarti, B., Orfila, C. & Sarkar, A. Oat protein: review of structure-function synergies with other plant proteins. *Food Hydrocoll.* **154**, 110139 (2024).

[83] Kew, B. et al. Transforming sustainable plant proteins into high performance lubricating microgels. *Nat. Commun.* **14**, 4743 (2023). 37550321 10.1038/s41467-023-40414-7 PMC10406910

[84] Pabois, O. et al. Benchmarking of a microgel-reinforced hydrogel-based aqueous lubricant against commercial saliva substitutes. *Sci. Rep.* **13**, 19833 (2023). 37985688 10.1038/s41598-023-46108-w PMC10662424

[85] Pabois, O. et al. Self-assembly of sustainable plant protein protofilaments into a hydrogel for ultra-low friction across length scales. *Commun. Mater.* **5**, 158 (2024). 39238825 10.1038/s43246-024-00590-5 PMC11371639

[86] Wang, M., Ettelaie, R. & Sarkar, A. Enzymatic hydrolysis of legume proteins: lessons on surface property outcomes. *Current Opin. Food Science*, 101259 (2024).

[87] Ployon, S. et al. The membrane-associated MUC1 improves adhesion of salivary MUC5B on buccal cells. Application to development of an in vitro cellular model of oral epithelium. *Arch. Oral Biol.* **61**, 149–155 (2016). 26580166 10.1016/j.archoralbio.2015.11.002

[88] Aasted, C. et al. Anatomical guidance for functional near-infrared spectroscopy: atlasviewer tutorial. *Neurophotonics* **2**, 020801 (2015). 26157991 10.1117/1.NPh.2.2.020801 PMC4478785

[89] Slinkard, K. & Singleton, V. L. Total phenol analysis: automation and comparison with manual methods. *Am. J. Enol. Viticult.* **28**, 49–55 (1977).

