# Supplementary material for: Unveiling plant protein astringency perception through neural and cellular responses
Source: Sci Rep. 2025 Dec 4;15:39184. doi: 10.1038/s41598-025-23836-9 (PMC12678544; doi:10.1038/s41598-025-23836-9)
Supplement: Supplementary file 1 — Supplementary Material 1 [file 41598_2025_23836_MOESM1_ESM.docx]

# Supplementary information

**Unveiling plant protein astringency perception through neural and cellular responses**

Ben Kew^1,2^**, Melanie Rose Burke^3***^, Markus Stieger^4^, Yunqing Wang^5^, Christine Boesch^2,5^, Melvin Holmes^1^, Anwesha Sarkar^1,2^*

^1^ Food Colloids and Processing Group, School of Food Science and Nutrition, University of Leeds, Leeds, LS2 9JT, UK

^2^ National Alternative Protein Innovation Centre (NAPIC), UK

^3^ School of Psychology, University of Leeds, Leeds, LS2 9JT, UK

^4^ Division of Human Nutrition and Health, Wageningen University, PO Box 17, 6700 AA Wageningen, The Netherlands

^5^ Nutritional Sciences and Epidemiology Group, School of Food Science and Nutrition, University of Leeds, Leeds, LS2 9JT, UK

Corresponding authors:

* Prof. Anwesha Sarkar

Email address: [A.Sarkar@leeds.ac.uk](mailto:A.Sarkar@leeds.ac.uk)

** Dr. Ben Kew

Email address: [B.J.Kew@leeds.ac.uk](mailto:B.J.Kew@leeds.ac.uk)

***Dr. Melanie Rose Burke

Email address: [m.r.burke@leeds.ac.uk](mailto:m.r.burke@leeds.ac.uk)


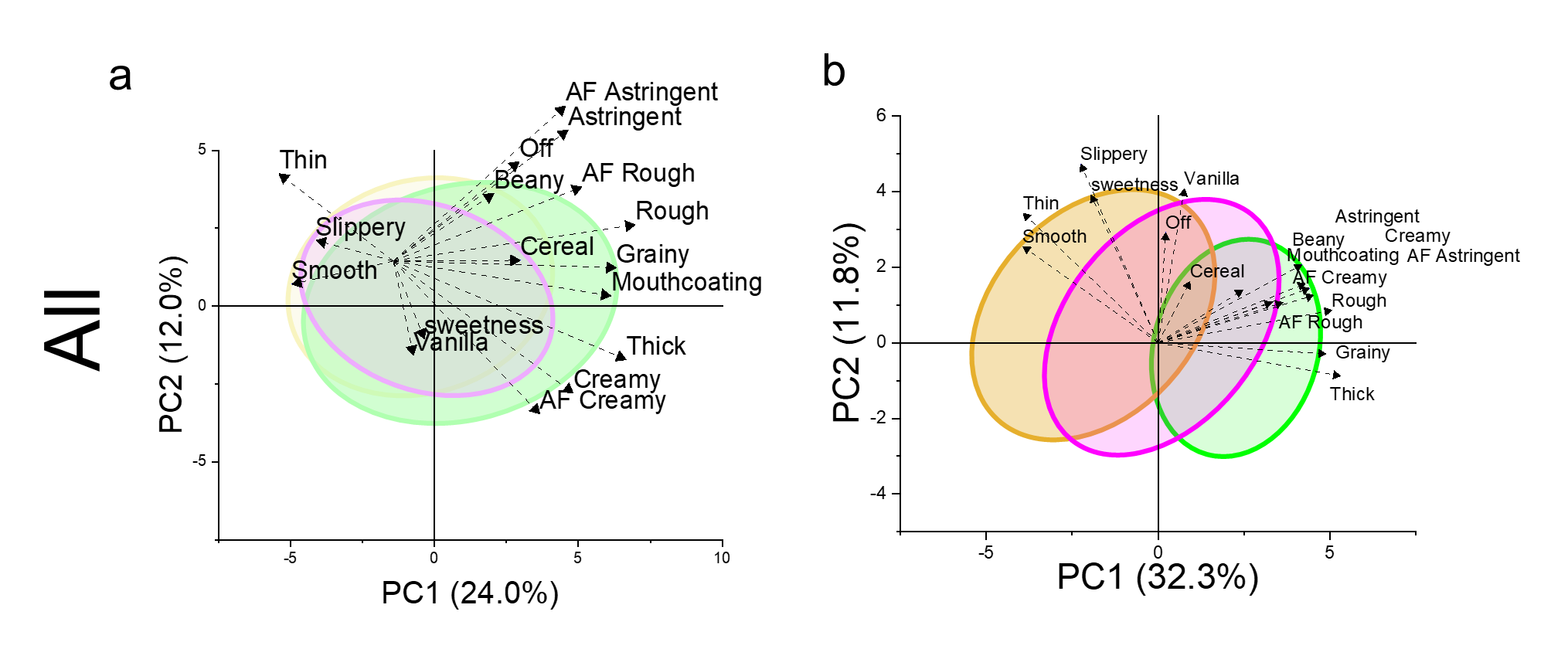


**Supplementary Fig 1| Overall sensory characteristics of model plant protein formulations individually at low and high concentrations.** Principle component analysis (PCA) of the taste, flavour, tactile, after feel (AF) attributes of model plant protein formulations containing plant proteins individually at (**a**) 5 wt% total protein (**b**) 15 wt% total protein, respectively. Results are presented from n=100 participants for the seventeen attributes tested. Ethical approval was obtained from the University of Leeds (MEEC 16-046 and PSYC-475) by the Faculty Ethics Committee, University of Leeds, UK.


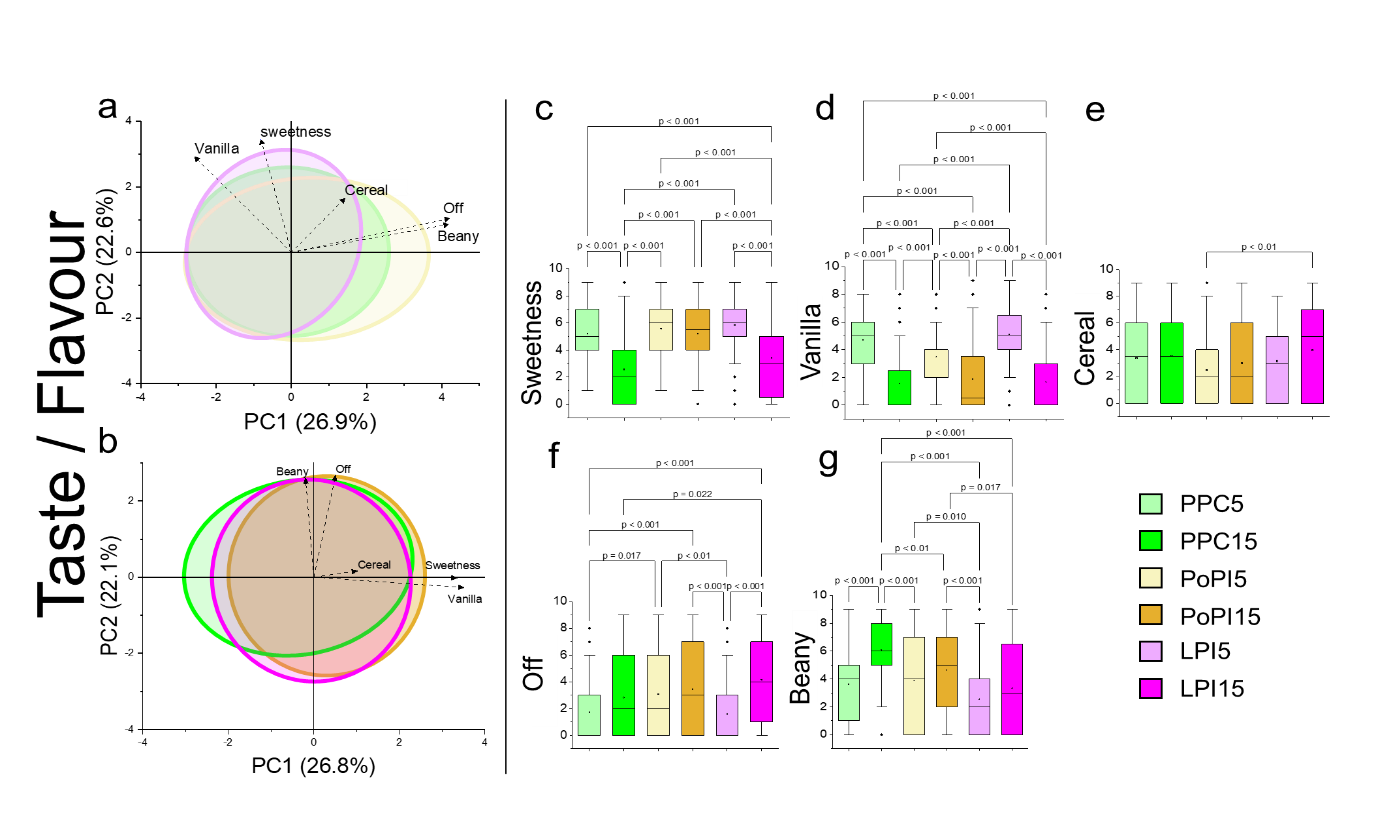


**Supplementary Fig 2| Taste and flavour characteristics of model plant protein formulations individually at low and high concentrations.** Principle component analysis (PCA) of the tactile attributes of model plant protein formulations containing plant proteins individually at (**a**) 5 wt% total protein (**b**) 15 wt% total protein, respectively. Taste and flavour ratings from Rate-All-That-Apply (RATA) are shown at 5 wt% and 15 wt% total protein for pea protein concentrate (PPC5, PPC15), potato protein isolate (PoPI5, PoPI15), lupin protein isolate (LPI5, LPI15) in (**c-g**) for five different tactile attributes. Results are presented as bar and whisker plots with interquartile range, minimum and maximum plotted (n=100 participants). Pairwise comparisons were made between type (PPC, PoPI, LPI) and protein concentration (5 – 15 wt% total protein) for each of the attributes applying Bonferroni correction. Ethical approval was obtained from the University of Leeds (MEEC 16-046 and PSYC-475) by the Faculty Ethics Committee, University of Leeds, UK.


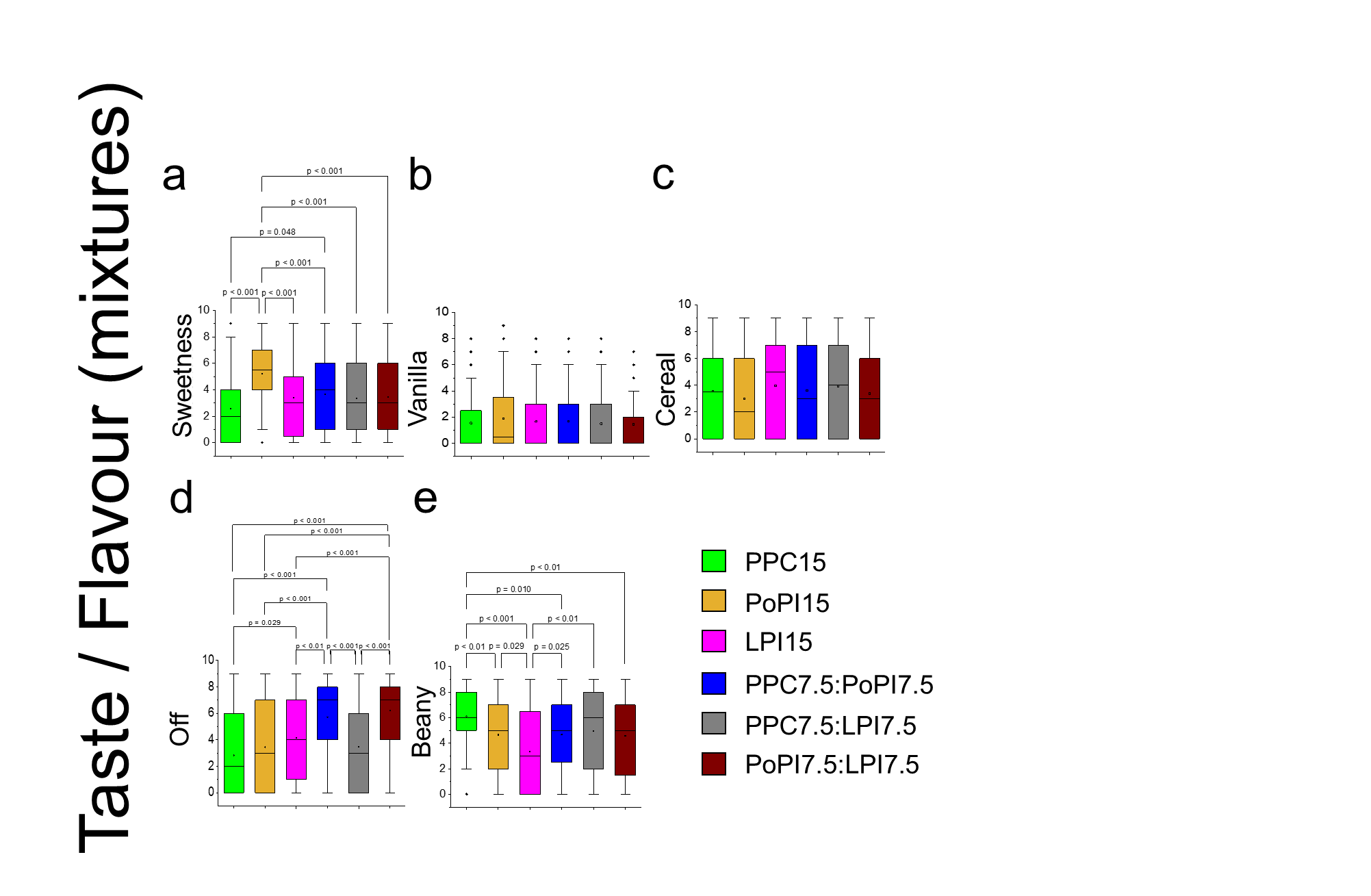


**Supplementary Fig 3| Taste and flavour characteristics of mixed protein formulations compared with individual proteins at higher protein levels**. Taste and flavour ratings from Rate-All-That-Apply are shown at 15 wt% total protein for pea protein concentrate (PPC15), potato protein isolate (PoPI15), lupin protein isolate (LPI15) and 1:1 binary protein mixtures (PPC7.5:PoPI7.5, PPC7.5:LPI7.5, PoPI7.5:LPI7.5) (**a-e**) obtained using Rate-All-That-Apply (RATA) in five different taste and flavour attributes. Results are presented as bar and whisker plots with interquartile range, minimum and maximum plotted (n=100 participants). Pairwise comparisons were made between type (PPC, PoPI, LPI, PPC-LPI, PPC-PoPI, PoPI-LPC) for each of the attributes applying Bonferroni correction. Ethical approval was obtained from the University of Leeds (MEEC 16-046 and PSYC-475) by the Faculty Ethics Committee, University of Leeds, UK.


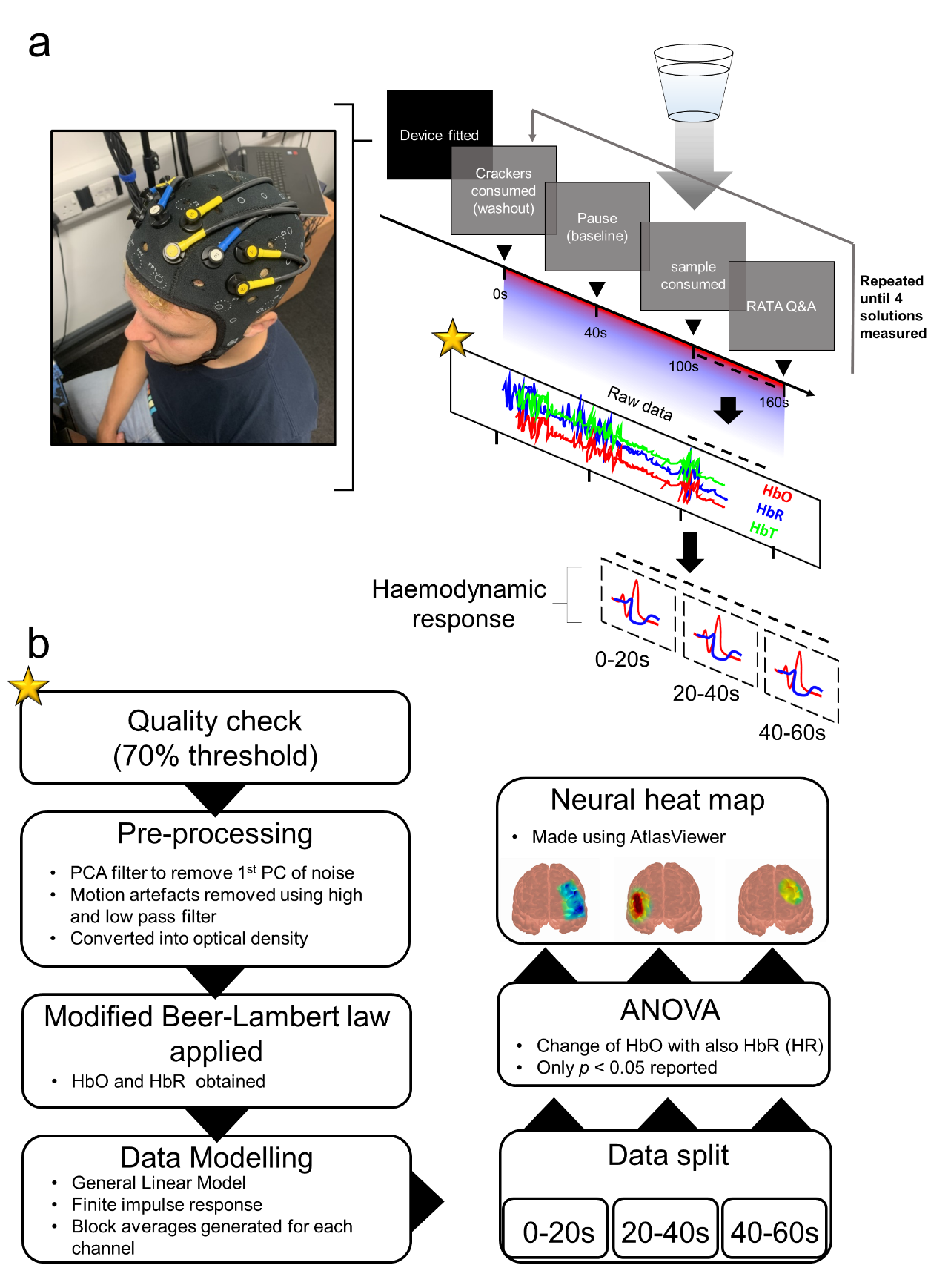


**Supplementary Fig 4| Experimental protocol of functional near-infrared spectroscopy (fNIRS**). (**a**) Visual image of participant wearing the cap and the overall procedure of fNIRS employed whilst consuming the plant proteins or tannic acid and the process of data collection, (**b**) the data filtering process and enhanced data quality assessment.


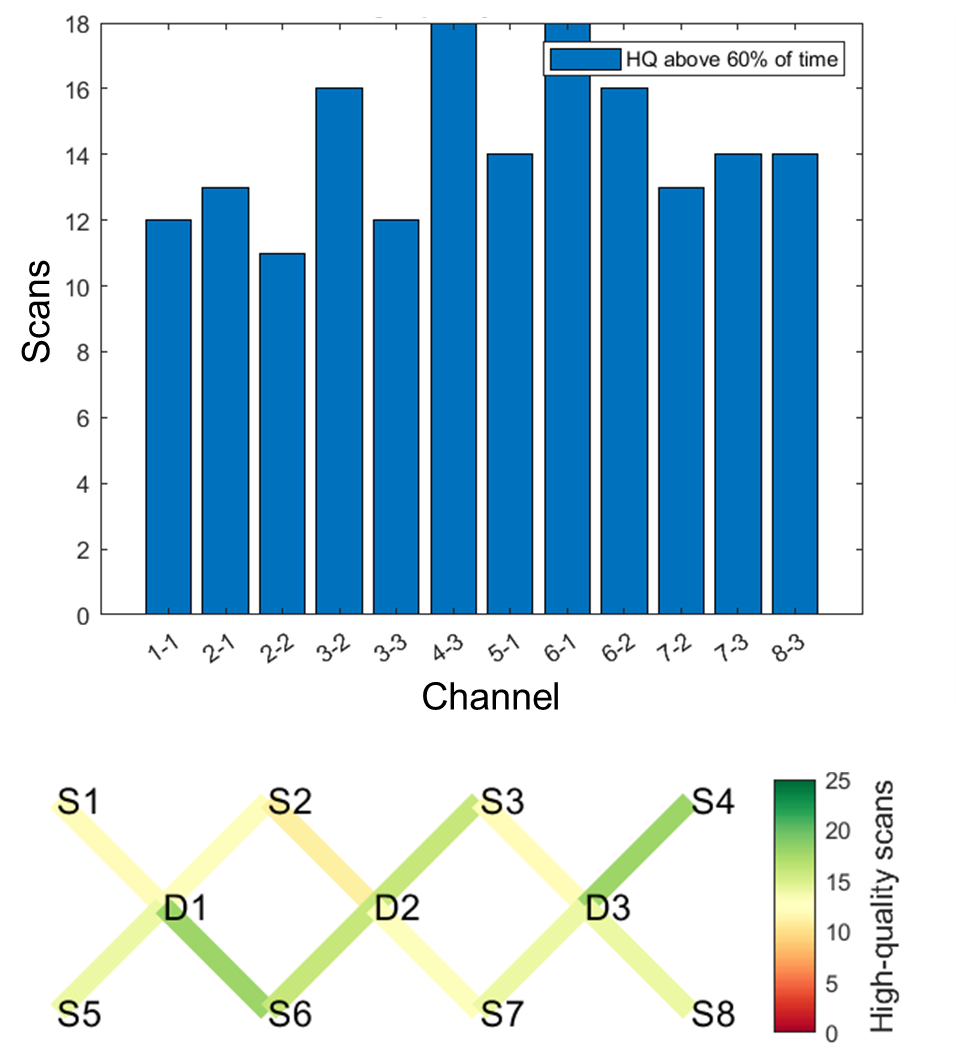


**Supplementary Fig 5| Quality checks used in data analysis of functional near infrared spectroscopy (fNIRS).** Quality analysis was performed using QTNirs in MatLab (<https://github.com/lpollonini/qt-nirs>). Channels were removed or trimmed that did not reach 70% threshold. Each channel secured good levels of quality and scans for accurate HR assessment.


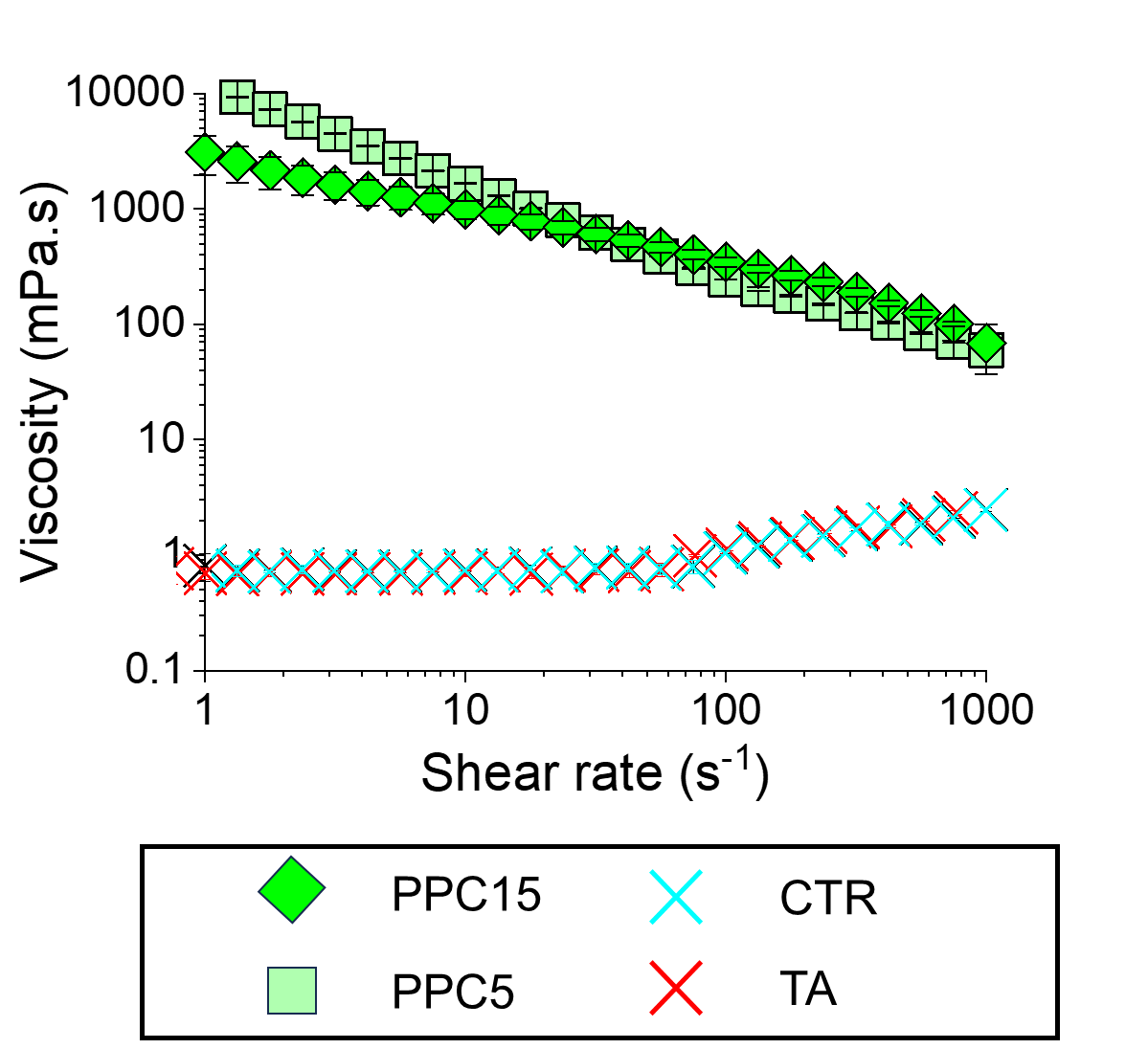


**Supplementary Fig 6| Flow curves of model formulations to standardise viscosity.** Apparent viscosity of 5 wt% total protein pea protein concentrate with xanthan gum (PPC5), 15 wt% total protein pea protein concentrate (PPC15), water (CTR) and 0.8 total wt% tannic acid (TA) in controlling viscosity of solutions when measuring neural response using functional near infrared spectroscopy (fNIRS). Shear rates were measured ramping up from 1 s^-1^ to 1000 s^-1^ at 37 ^o^C with plots as means of three measurements on triplicate samples (n = 6 x 3) with error bars representing standard deviations.


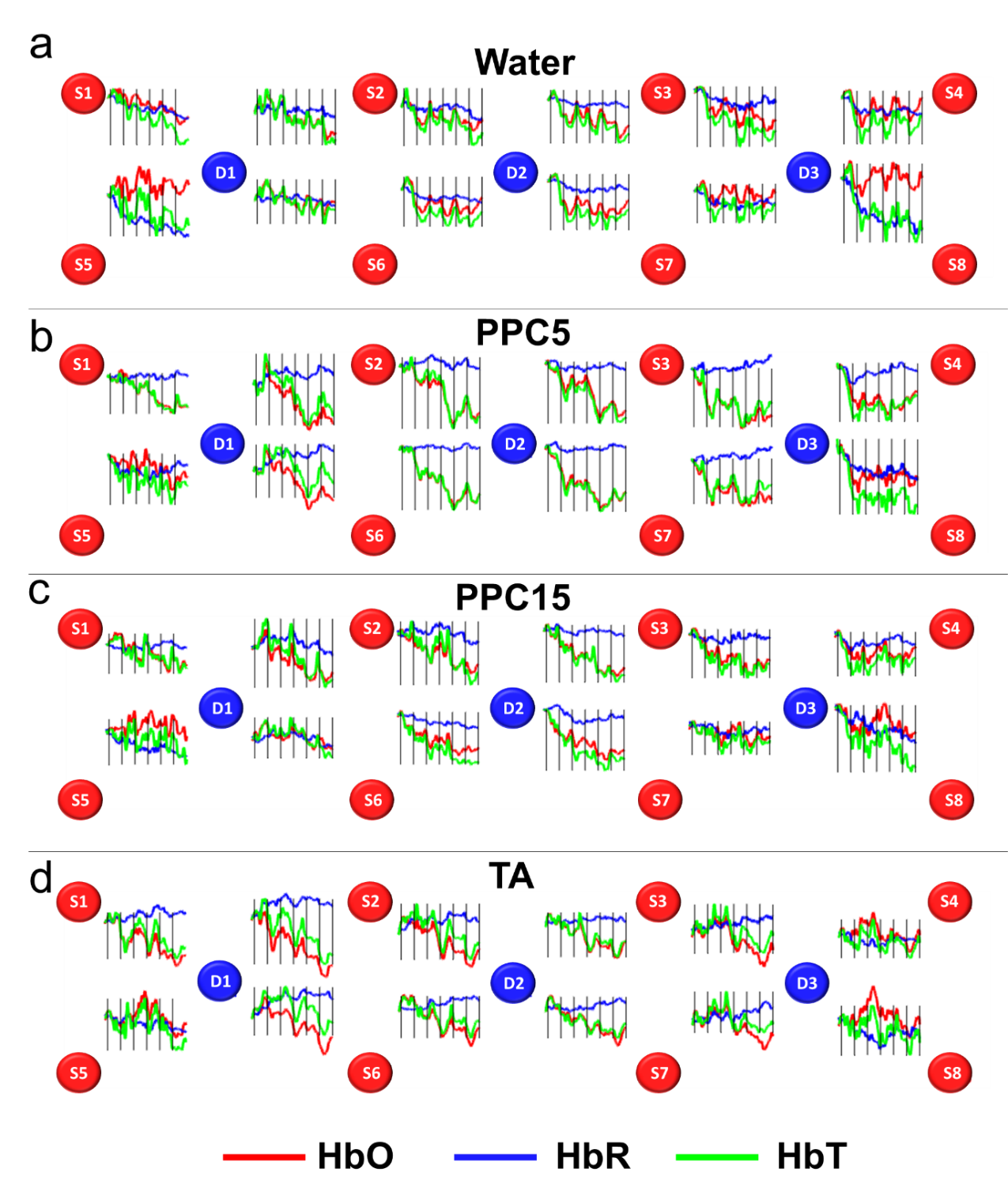


**Supplementary Fig 7| Total haemodynamic response analysed using** **functional near infrared spectroscopy (fNIRS).** Average total haemodynamic response including oxygenated haemoglobin (HbO), deoxygenated haemoglobin (HbR) and total haemoglobin (HbT) from 0-60s when consuming (**a**) non-astringent water (Water), (**b**) low astringent, viscosity matched 5 wt% total protein pea protein concentrate (PPC5), (**c**) 15 wt% pea protein concentrate (PPC15) and (**d**) 0.8 wt% tannic acid (TA).


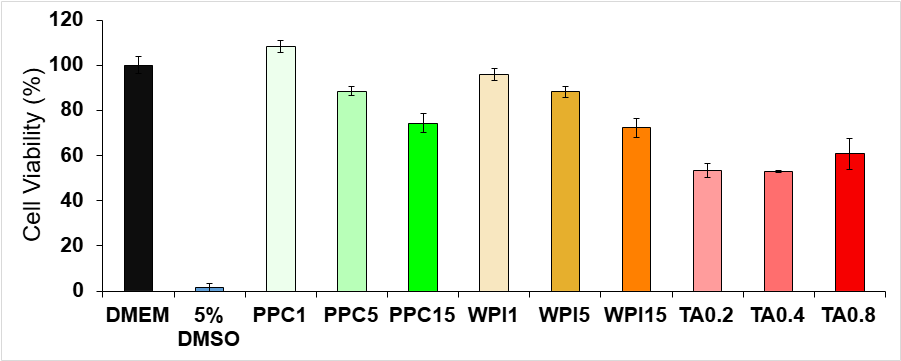


**Supplementary Fig 8|** **Cytotoxicity of model protein formulations and controls.** Cell viability (%) as compared to medium control (Dulbecco's Modified Eagle Medium (DMEM) of 1-15 wt% total protein pea protein concentrate (PPC1, PPC5, PPC15), 1-15 wt% total protein whey protein isolate (WPI1, WPI5, WPI15), 0.2-0.8 wt% tannic acid (TA0.2, TA0.4, TA0.8). Results are plotted as means and standard deviations of 6 repeat measurements on triplicate samples (n = 6 x 3) with error bars representing standard deviations.

**Supplementary Fig 9|** **Total phenolic content (Gallic acid equivalent) of proteins.** Total phenolic content of whey protein isolate (WPI) and pea protein concentrate (PPC) powder extracts. Three independent replicates were ran for each sample where no significant difference were found in total phenolic content between proteins (*P* > 0.05).

**Supplementary Table 1|** Overview of sensory terms and definitions used in Rata-All-That-Apply (RATA).

| **Category** | **RATA Terms** | **Definitions** |
| --- | --- | --- |
| Taste | Vanilla flavour | The degree to which the sample tastes like vanilla |
|  | Off flavour | The degree to which the sample tastes undesirable/harsh in flavour |
|  | Cereal flavour | The degree to which the sample tastes like hay/straw/grains |
|  | Sweetness | Sweet sensation from sucrose, sweetener |
|  | Beany flavour | Bean, pea like flavour |
| Texture | Creamy | The degree to which the sample gives a silky, rich, full mouthfeel. |
|  | Slippery | The degree to which sample can slide easily in the mouth |
|  | Grainy | The presence of granules, particles in the mouth |
|  | Smooth | Homogenous; absence of lumpiness and the sample flows easily in the mouth. |
|  | Thick | The thickness of the sample; the amount of force needed to make the sample flow or deform in the mouth. |
|  | Thin | The lack of force needed to allow the sample to flow in the mouth |
|  | Rough | A drying, lack of lubrication, sensation in the mouth |
|  | mouthcoating | The feeling that a layer of the sample remains behind in the mouth and palate after swallowing. |
|  | Astringent | Intense puckering, mouth-drying, saliva-binding sensation |
| After-Feel (AF) | Creamy | The degree to which the sample gives a silky, rich, full mouthfeel. |
|  | Rough | A drying, lack of lubrication, sensation in the mouth |
|  | Astringent | Intense puckering, mouth-drying, saliva-binding sensation |

**Supplementary Table 2|** Motor neurological institute (MNI) coordinate system with corresponding Brodmann (BA) areas and location of source and detector nodes used.

| Source | Detect | MNI | Brain Location |
| --- | --- | --- | --- |
| 1 | 1 | 20, 25, 44 | Frontal Eye Fields (BA8) |
| 2 | 1 | 11, 28, 36 | Frontal Eye Fields (BA8) |
| 5 | 1 | 36, 32, 27 | Right DLPFC (BA9) |
| 6 | 1 | 16, 46, 29 | Right DLPFC (BA9) |
| 2 | 2 | 2 38 33 | Right DLPFC (BA9) |
| 3 | 2 | -11, 44, 42 | Left FEF (BA8) |
| 6 | 2 | 8, 64, 39 | Right DLPFC (BA9) |
| 3 | 3 | -29, 44, 47 | Left DLPFC (BA9) |
| 4 | 3 | -38, 26, 37 | Left DLPFC (BA9) |
| 7 | 3 | -26, 52, 30 | Left aPFC (BA10) |
| 8 | 3 | -51, 44, 30 | Left aPFC (BA10) |
| 7 | 2 | -16, 65, 36 | Left DLPFC (BA9) |

**Supplementary Table 3|** Data values of the block averaged overall neural response using functional near-infrared spectroscopy (fNIRS) between 0-60 s when consuming non-astringent water (Water), low astringent, viscosity matched 5 wt% total protein pea protein concentrate (PPC5), 15 wt% pea protein concentrate (PPC15) and 0.8 wt% tannic acid (TA). Beta values are included from T-stat (n=34 participants). A positive value reflects increase in HR compared to baseline whilst a negative value reflects decreases in HR. Individual subject data was subjected to a mixed effects group ANOVA where *p* < 0.05 reflects a statistically significant difference.

| Time (s) | Condition | Source | Detector | Beta | Tstat | P value | Power |
| --- | --- | --- | --- | --- | --- | --- | --- |
| 0 – 20 | PPC15 | 5 | 1 | 5.376 | 4.635 | 0.001 | 0.208 |
|  | PPC15 | 2 | 2 | -1.754 | -2.360 | 0.020 | 0.324 |
|  | PPC5 | 5 | 1 | 1.882 | 2.486 | 0.015 | 0.318 |
|  | TA | 4 | 3 | -1.907 | -2.151 | 0.034 | 0.272 |
|  | TA | 8 | 3 | -3.071 | -3.445 | 0.001 | 0.270 |
| 20-40 | TA | 5 | 1 | 4.131 | 3.034 | 0.003 | 0.211 |
|  | PPC5 | 4 | 3 | 4.991 | 4.192 | 0.001 | 0.241 |
|  | PPC5 | 8 | 3 | 4.619 | 3.987 | 0.001 | 0.248 |
| 40-60 | TA | 3 | 3 | 2.322 | 2.128 | 0.037 | 0.266 |
